# Chemical Modification of *Thermomyces lanuginosus* Lipase and *Myceliophthora thermophila* Laccase Using Dihydrazides: Biochemical Characterization and In Silico Studies

**DOI:** 10.3390/ijms262211094

**Published:** 2025-11-16

**Authors:** Juan S. Pardo-Tamayo, Maria Camila Muñoz-Vega, Oscar L. Alférez, Evelyn L. Guerrero-Tobar, Chonny Herrera-Acevedo, Ericsson Coy-Barrera, César A. Godoy

**Affiliations:** 1 Laboratorio de Investigación en Biocatálisis y Biotransformaciones (LIBB), Grupo de Investigación en Ingeniería de los Procesos Agroalimentarios y Biotecnológicos (GIPAB), Department of Chemistry, Universidad del Valle, Cali 760001, Colombiamoreno.oscar@correounivalle.edu.co (O.L.A.); evelyn.guerrero@correounivalle.edu.co (E.L.G.-T.); 2 Department of Chemical Engineering, Universidad ECCI, Bogotá 111311, Colombia; cherreraa@ecci.edu.co; 3 Bioorganic Chemistry Laboratory, Facultad de Ciencias Básicas y Aplicadas, Universidad Militar Nueva Granada, Cajicá 250247, Colombia; ericsson.coy@unimilitar.edu.co

**Keywords:** enzyme chemical modification, laccase, lipase, dihydrazides, amination, kinetic parameters, molecular dynamics

## Abstract

Chemical modification is a valuable strategy for tuning enzyme functionality by introducing new reactive groups without disrupting the overall fold. Conventional amination using ethylenediamine (EDA) is effective, but the resulting modified proteins show limited reactivity for conjugation at neutral pH, and the modifier itself poses safety concerns due to its volatility and corrosive nature. Dihydrazides, in contrast, offer a safer and more versatile alternative: they operate through the same carboxyl-activation mechanism while enabling systematic investigation of chain-length effects. In this study, *Thermomyces lanuginosus* lipase (TLL) and *Myceliophthora thermophila* laccase (MTL) were modified using dihydrazides with different alkyl chain lengths (carbonyl (CZ), oxalyl (OX), succinyl (SC), and adipic (AA)), and compared to EDA-modified and unmodified enzymes to evaluate their effects on catalytic performance. Hydrazide-modified variants exhibited enhanced catalytic performance, reaching up to 2.5-fold (TLL-CZ) and 4.2-fold (MTL-AA and MTL-OX) higher efficiencies than unmodified and EDA-modified enzymes. Notably, AA provided the most consistent improvement across both enzymes (1.3-fold in TLL and the best in MTL). Molecular dynamics and docking analyses supported these findings, linking increased flexibility (higher RoG and RMSF) with higher k_cat_, and changes in substrate binding with lower k_m_. Overall, hydrazide-based modification broadens the spectrum of enzyme variants attainable through amination, while offering safer procedures, thus representing an alternative that overcomes the limitations of using EDA as a conventional aminating agent.

## 1. Introduction

Biocatalysis has gained importance in industry due to its high specificity, mild operating conditions, and lower environmental impact compared to conventional catalysts, aligning with green chemistry principles [1]. Lipases and laccases are among the enzymes of biotechnology interest, valued for their versatility and broad substrate specificity. Lipases catalyze the hydrolysis of triglycerides and are applied in various sectors, including food, detergents, biofuels, and fine chemicals [2,3]. Laccases, on the other hand, oxidize phenolic substrates using molecular oxygen and are employed in textiles, paper, cosmetics, and environmental remediation [4,5,6]. Nonetheless, improving enzyme stability and activity remains a significant challenge for industrial applications. Strategies to address this issue encompass genetic methods such as directed evolution [7] and physicochemical modifications [8,9], have been developed. Notably, physicochemical modifications are becoming increasingly efficient and controllable [6,8,9], allowing for improved biocatalytic parameters and enhanced stability under specific conditions, such as variations in pH, temperature, and solvent presence [9]. These strategies can be combined to develop advanced enzyme systems, including plurienzymes with multiple active sites [10,11] or immobilized enzyme derivatives, which often exhibit superior activity and stability [6,9,12].

Surface amination of proteins using ethylenediamine (EDA) and carbodiimide reagents (commonly EDC) is among the most widely applied chemical modifications. The incorporation of primary amino groups introduces positive charges, often improving enzyme stability under varying pH, temperature, and solvent conditions [6,9]. On the other hand, EDA serves as a bifunctional linker that enables further derivatization [12], stabilization via methods such as cross-linked enzyme aggregates (CLEAs) [13], and covalent immobilization on electrophilic supports like glyoxyl- or epoxide-agarose [3,14,15]. EDA-modified enzymes have demonstrated improved activity (e.g., in *Horseradish* peroxidase HRP [16]) and enhanced reactivity toward aldehydes and epoxides, facilitating covalent bioconjugation between different proteins [16].

However, EDA poses several challenges since it is volatile, irritating, corrosive, and susceptible to oxidation in solution, which limits its safe handling and application [17]. Dihydrazides are safer alternatives, offering similar reactivity toward electrophiles, improved stability of the bond formed (hydrazones), and neutral nucleophilic character at physiological pH [9]. These groups have been used in peptide synthesis via native chemical ligation [18], in enzymatic modifications [8,9], and in studies of post-translational modifications. Adipic acid dihydrazide (AA), in particular, has been applied in protein–protein bioconjugation and HRP modification for improved hormone detection [16,19]. Despite their promising reactivity and safer profile, to our knowledge, the impact of dihydrazide-based modifications, particularly regarding the influence of alkyl chain length, on enzyme catalytic behavior and structure has not been thoroughly explored.

In this context, molecular dynamics (MD) simulations offer an approach to explore these effects at the atomic level. By modeling the physical movements of atoms and molecules over time, MD enables the analysis of structural, dynamic, and thermodynamic properties of biomolecules [20]. This includes insights into how proteins respond to external stimuli or covalent modifications, revealing conformational changes, alterations in flexibility, and shifts in folding or stability. Previous studies have demonstrated the utility of MD in evaluating chemical modifications on enzyme surfaces [20], although its routine application in biocatalyst design remains limited.

In this study, we hypothesized that chemical modification of enzymes using dihydrazides of different chain lengths could modulate their catalytic behavior and alter their structural stability by changing the microenvironment around the modified Glu and Asp residues. This strategy aimed to extend the applicability of conventional EDC-mediated modification—typically employing EDA-based amination—by introducing tunable, non-charged linkers suitable for future bioconjugation [19], while replacing EDA with a modifier that is less corrosive, odorless, and safer to handle.

To test this hypothesis, two model enzymes—*Thermomyces lanuginosus* lipase (TLL) and *Myceliophthora thermophila* laccase (MTL)—were systematically modified with dihydrazides of different chain lengths (carbonyl, oxalic, succinic, and adipic) and characterized through a combined experimental–computational approach. The modified enzymes were kinetically analyzed using the Michaelis–Menten model and evaluated for their stability in organic solvents and at elevated temperatures. Complementary molecular dynamics (MD) simulations were performed on in silico-modified structures to correlate it with the experimental results. Finally, the modified enzymes were evaluated in biotechnologically relevant reactions—fatty ester synthesis (a key component of biodiesel) for TLL and indigo carmine biodegradation for MTL—as model applications to explore their functional viability.

## 2. Results and Discussion

### 2.1. Enzyme Chemical Modification

**Scheme 1:**
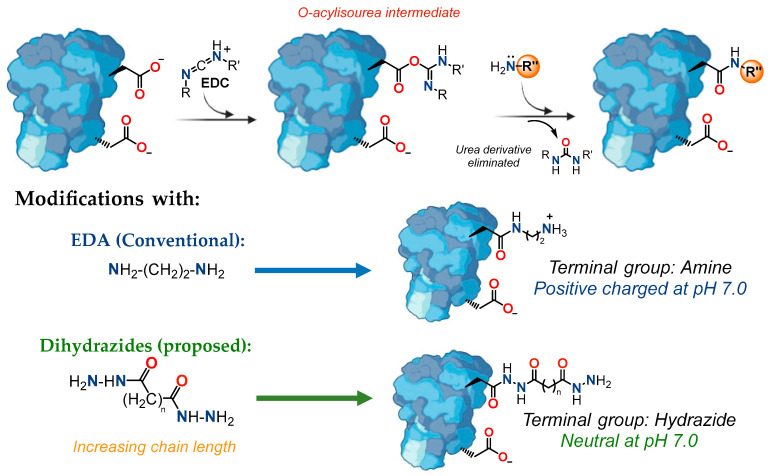
Overview of the EDC-mediated coupling between enzyme carboxyl groups and different modifiers [9,26]. The scheme highlights the formation of the O-acylisourea intermediate (top) and the resulting enzyme–modifier conjugates for the conventional cationic reagent EDA (positively charged at pH 7.0) and for neutral dihydrazides of different chain lengths (uncharged at pH 7.0) (bottom). Image created with BioRender.com.

Enzymatic modifications were initially conducted using a solid-phase modification with EDA and adipic acid dihydrazide (AA), based on a previously reported protocol employing 0.5 M modifier and 10 mM EDC [3,12,21]. Enzymes were immobilized on Lewatit^®^ VPOC1600, Q-Sepharose^®^, and PEI-agarose supports (Table S1). TLL showed ~20% recovery in terms of activity and protein on the hydrophobic support Lewatit^®^, likely due to favorable interactions with its binding domain [22,23], and the poor interference of these types of interactions in the modification. MTL could not be immobilized on this support. In contrast, immobilization on ion-exchange supports resulted in poor recovery (<12% in activity and <6%) and no desorption in the case of PEI-agarose, possibly due to covalent interactions promoted by EDC [24,25]. This outcome is consistent with the intrinsic reactivity of EDC-activated carboxyl groups (from Asp and Glu residues) toward nucleophilic nitrogen-based moieties on the support (Scheme 1, top). That could be expected due the same versatility was exploited here to attach two different types of modifiers—EDA or dihydrazides—through analogous coupling chemistry but yielding distinct terminal groups (Scheme 1, bottom).

Due to these constraints, a liquid-phase modification strategy was adopted [6]. Preliminary assays varying EDC concentration showed that the extent of modification increased with EDC levels (Table S2), consistent with the mechanism of carbodiimide-mediated coupling, where the rate-limiting step involves coupling between EDC and protein carboxyl groups to form the *O-*acylisourea intermediate (Scheme 1) [27]. However, enzymatic activity remained low (<23%), likely due to modification of catalytic residues or active site obstruction, effects that are typically prevented when using hydrophobic supports [21]. In contrast, protein recovery was improved to ~39% for EDA and ~72% for AA, indicating that AA-modified versions were easier to purify than aminated ones, possibly due to the reduction in surface charge when dihydrazides were used (Scheme 1). This represents an operational advantage, along with the procedural simplification and improved safety of using hydrazides instead of EDA, as hydrazides facilitate pH adjustment and do not release gases [17,28].

To mitigate the loss of activity observed during modification, CTAB was added to the reaction medium. This cationic surfactant is known to stabilize the open, active form of lipases by reducing intermolecular aggregation and shielding catalytic residues from undesired modification [24,29,30,31]. Although this conformational effect was not directly validated in this work, its functional consequence was clearly evidenced by the marked increase in recovered activity and modification yield. At low CTAB concentrations (0.00–0.01%), activity recovery rose moderately, in the best case for TLL-AA, from 21.9% to 59.8%, while at 0.10% CTAB it exceeded 100% in some cases, with modification yields reaching ≈66%, compared to <23% and ≈40% in its absence (Table S3). This improvement is consistent with the stabilization of the open conformation, which prevents irreversible inactivation and modification of catalytic residues. The response plateaued at higher CTAB levels, likely near the CMC [29], indicating an optimal balance between reactive site exposure and preservation of catalytic activity.

Once the best tested modification conditions were established, both TLL and MTL were chemically modified using four dihydrazides of varying alkyl chain lengths: carbonyl dihydrazide (CZ), oxalic dihydrazide (OX), succinic dihydrazide (SC), and adipic dihydrazide (AA). Although AA had been previously reported for enzyme modification [16,19], the effect of dihydrazide chain length on the same enzyme had not been systematically evaluated and compared across different enzyme types. Ethylenediamine (EDA) modifier was included as a reference modifier due to its established role in enhancing enzyme reactivity and enabling further functionalization [32]. Table 1 summarizes the percentage of surface modification, protein and activity recovery, and specific activity (IU/mg) obtained for each derivative under standardized conditions.

It is important to highlight that most modifications positively affected enzymatic activity, with the exceptions being TLL modified with EDA and OX, and MTL modified with CZ. Remarkably, TLL-AA showed nearly a twofold activity increase, while MTL-AA exhibited an approximately sixfold increase. Overall, the modification percentages ranged between 45–66% for TLL and 40–54% for MTL (excluding MTL-CZ). SDS-PAGE analysis (Supplementary Table S4, Figures S1 and S2) revealed that EDA-modified enzymes did not show multimeric forms, likely because the surface charges introduced hinder intermolecular crosslinking. In contrast, several TLL variants modified with hydrazides formed dimeric and trimeric species, probably covalent, which may partly account for their reduced activity in some cases.

### 2.2. Biocatalytic Characterization of Modified Enzymes

#### 2.2.1. TLL Surfactant Activation Studies: Impact of Chemical Modifications

**Figure 1:**
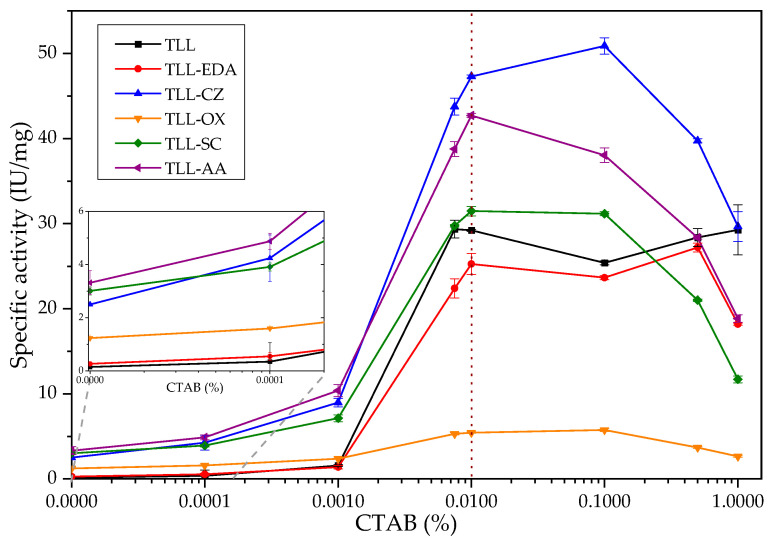
Activation curves showing specific hydrolytic activity (IU/mg) versus CTAB concentration (w/v%) for unmodified TLL (black), TLL-EDA (red), TLL-CZ (blue), TLL-OX (orange), TLL-SC (green), and TLL-AA (purple). CMC is shown as a vertical dashed line at 0.0100%.

Certain lipases, TLL included, possess a lid domain that controls access to the active site, transitioning from inactive (closed or multimeric) to active conformation upon contact with hydrophobic interfaces (like an oil droplet or micelle), a phenomenon known as interfacial activation [33]. Surfactants such as CTAB can mimic this effect, as was mentioned in Section 2.1, stabilizing the active monomeric conformation [2,29,34], a property that underlies their widespread use in biotechnological formulations. This feature was exploited here to evaluate whether the chemical modifications altered the enzyme’s responsiveness to interfacial activation and, consequently, the equilibrium between open and closed forms. For that purpose, activation curves (Figure 1) were generated by measuring the specific hydrolytic activity (IU/mg) across increasing CTAB concentrations (w/v%).

All TLL variants, including those modified with EDA and the four dihydrazides (CZ, OX, SC, and AA), exhibited similar CTAB-induced activation patterns, indicating that their conformational equilibrium remained responsive to surfactant-mediated activation. This preservation of the activation behavior suggests that the chemical modifications did not disrupt the natural lid-controlled mechanism of TLL but rather modulated its amplitude or intensity between variants. Maximal activity was observed near the CMC (~0.01%), declining at higher concentrations, consistent with the typical inhibition caused by micelle formation [29,35,36]. Among the variants, TLL-CZ reached the highest activity (~50 IU/mg at 0.10% CTAB), nearly doubling that of the unmodified enzyme (TLL) and TLL-EDA (25–29 IU/mg), suggesting a favorable effect of CZ on lid dynamics and catalytic performance.

**Figure 2:**
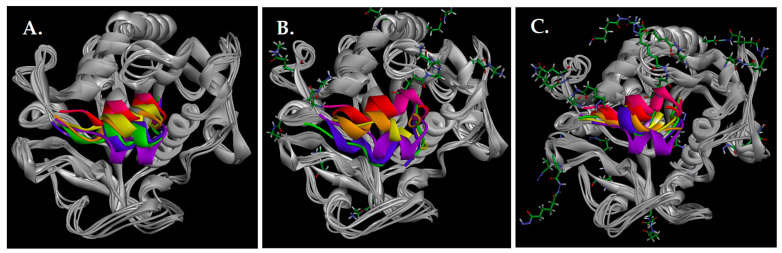
Lid flexibility of TLL (**A**), TLL-EDA (**B**), and TLL-AA (**C**) was assessed via 100-ns MD simulations starting from the open-state conformation. Snapshots at key time points—red (0 ns), orange (18 ns), yellow (43 ns), blue (92 ns), green (100 ns)—were superimposed onto the crystal structures of TLL in its open (pink, PDB:1DTE) and closed (purple, PDB:1DT5) conformations to illustrate the progression toward closure.

Molecular dynamics (MD) simulations (100 ns) were conducted on models of open-state TLL modified with either hydrazide or EDA (models were obtained using the methodology detailed in Section 3.12) to further examine the impact of chemical alterations on TLL conformational behavior (Figure 2 and Figure S3). The spontaneous closure observed in the MD simulations of the unmodified enzyme aligns with the natural open–closed equilibrium of lipases, which surfactants such as CTAB are known to shift toward the open, more catalytically active state [2]. Snapshots revealed that unmodified TLL underwent a gradual lid closure, reaching a near-closed conformation by 92 ns (Figure 2A). This transition occurred rapidly (43 ns) and was more pronounced in TLL-EDA (Figure 2B), aligning with its reduced response to CTAB. In contrast, dihydrazide-modified variants—particularly TLL-AA (Figure 2C)—exhibited reduced lid movement, consistent with a stabilization of the open, catalytically active form inferred from the activation curves. This aligns with the higher activity observed for TLL-AA at low CTAB concentrations (see inset in Figure 1). The lower activity of TLL-OX may be attributed to its higher proportion of inactive dimers (34%, Table S4), which are known to hinder substrate access to the catalytic site [37].

When analyzing the effect of chain length on lid mobility and on the stabilization of the enzyme’s active conformation, it was observed that the chemically modified variants showing the highest specific activities upon CTAB addition—CZ, AA, and SC (with carbon chain lengths of 1, 6, and 4, respectively)—did not follow a clear trend with respect to chain length (Figure 1). Nonetheless, an inspection of the MD trajectories focused on the lid region (Figure 2) indicated that variants bearing bulkier substituents tended to display smaller displacements from the fully open starting state within the 100-ns window. This behavior is consistent with a partial stabilization of open or semi-open conformations, which would preserve catalytic accessibility (Figure 2, Figures S3 and S4). Conversely, variants with shorter substituents—particularly EDA and OX—showed faster (after approximately 18 ns) progression toward more closed-like arrangements (Figure 2B), suggesting that linkers with two carbons of length may facilitate lid compaction. Interestingly, the CZ variant behaved differently: although its RMSD increased rapidly from the initial open conformation, the final structure at 100 ns (Figure S3A, blue trace) did not correspond to the closed state observed for the other variants. Consistent with the experimental data (Figure 1, blue line), this alternative conformation appears to be more catalytically active. A more comprehensive analysis of chain-length effects on global flexibility and catalytic performance, including RMSD/RMSF correlation with kinetic parameters, is presented in Section 2.2.2.

#### 2.2.2. Kinetic Studies for TLL and Its Variants

**Scheme 2:**
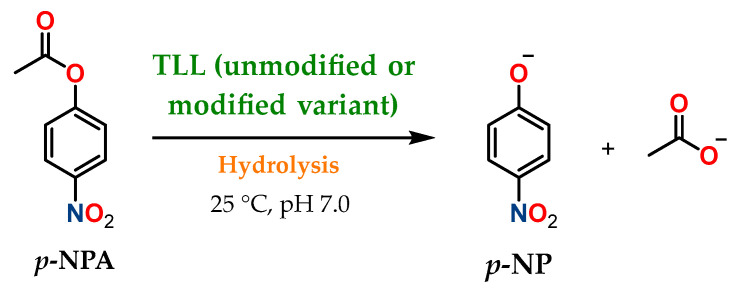
Hydrolysis of *p*-nitrophenyl acetate (*p*-NPA) catalyzed by TLL and its chemically modified variants. The release of *p*-nitrophenol (*p*-NP) as is described in Section 3.2 [29,38].

Although TLL is a widely used biocatalyst, including applications beyond lipid substrates, surprisingly few kinetic studies have employed *p*-NPA (Scheme 2). This gap makes the present results particularly valuable for benchmarking and for exploring TLL’s potential in small-molecule transformations, such as the synthesis of pharmaceutical building blocks [2]. Michaelis–Menten kinetic parameters were determined for unmodified and modified TLL variants using *p*-NPA hydrolysis at 25 °C and pH 7.0 with 0.01% CTAB (Scheme 2) (Section 3.9). Results are shown in Table 2 and Figure S5.

The unmodified enzyme exhibited a k_cat_ of 70.1 s^−1^ and a k_m_ of 1.3 mM, resulting in a catalytic efficiency of 55.6 mM^−1^ s^−1^. Among the modified enzymes, TLL-CZ showed the greatest improvement in both parameters, with k_cat_ = 98.0 s^−1^, k_m_ = 0.7 mM. Although the individual changes in k_cat_ and k_m_ were relatively modest, their combined effect yielded a marked increase in catalytic efficiency (k_cat_/k_m_ = 138.0 mM^−1^ s^−1^). TLL-SC and TLL-AA also displayed enhanced performance, with efficiencies (k_cat_/k_m_) of 90.5 and 75.0 mM^−1^ s^−1^, respectively. In contrast, TLL-EDA and especially TLL-OX showed reduced turnover (k_cat_) and efficiency (k_cat_/k_m_), suggesting detrimental effects from these modifiers. The results underscore that hydrazide-based modifications—specifically CZ, SC, and AA—can improve TLL catalytic efficiency, while EDA and OX may detrimentally influence activity; this would presumably be attributable to lid dynamics or aggregation-related factors, as discussed in the previous section.

**Figure 3:**
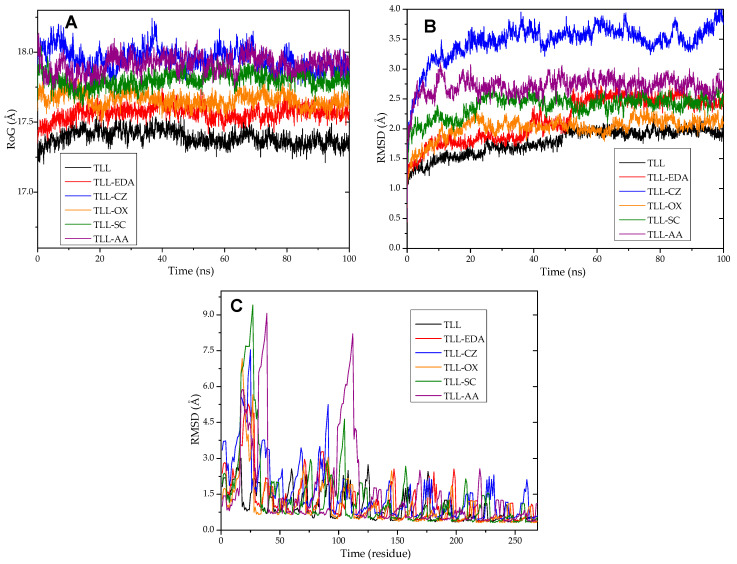
(**A**) Radius of gyration (RoG), (**B**) Root mean square deviation (RMSD), and (**C**) root mean square fluctuation (RMSF) plots of the open-state TLL and modified versions produced after 100-ns molecular dynamics simulations. TLL (black line, PDB:1DTE); TLL-AA (purple line); TLL-OX (orange line); TLL-EDA (red line); TLL-CZ (blue line), and TLL-SC (green line).

To further understand how chemical modifications affect TLL structure and function, 100 ns molecular dynamics simulations were performed using open-state models. The MD 100 ns simulations showed that all modified enzymes exhibited increased structural flexibility compared to unmodified TLL, as reflected by higher radius of gyration (RoG) and root mean square deviation (RMSD) values (Figure 3A,B).

The increase in RoG values suggests a less compact globular structure (Figure 3A), while RMSD (Figure 3B) provides an extent of the structural deviation from the initial conformation, reflecting the overall stability and flexibility of the enzyme during the simulation. Among the variants, TLL-CZ exhibited the highest RoG (~18.2 Å) and RMSD values (~3.5–4.0 Å), both of which increased steadily throughout the simulation, without reaching a plateau (Figure 3A,B), suggesting significant conformational mobility and incomplete structural stabilization within the 100-ns window. TLL-AA and TLL-SC also displayed increased RoG and intermediate RMSD values, indicating reduced compactness and greater conformational mobility—possibly likely sufficient to affect catalytic properties without compromising stability. In contrast, TLL-OX and TLL-EDA maintained values closer to those of the unmodified enzyme (~17.4–17.8 Å RoG and ~2.0–2.5 Å RMSD), indicating milder effects (Figure S6, Supporting Materials). However, excessive expansion—as observed in TLL-CZ—could also compromise stability if not carefully controlled.

**Figure 4:**
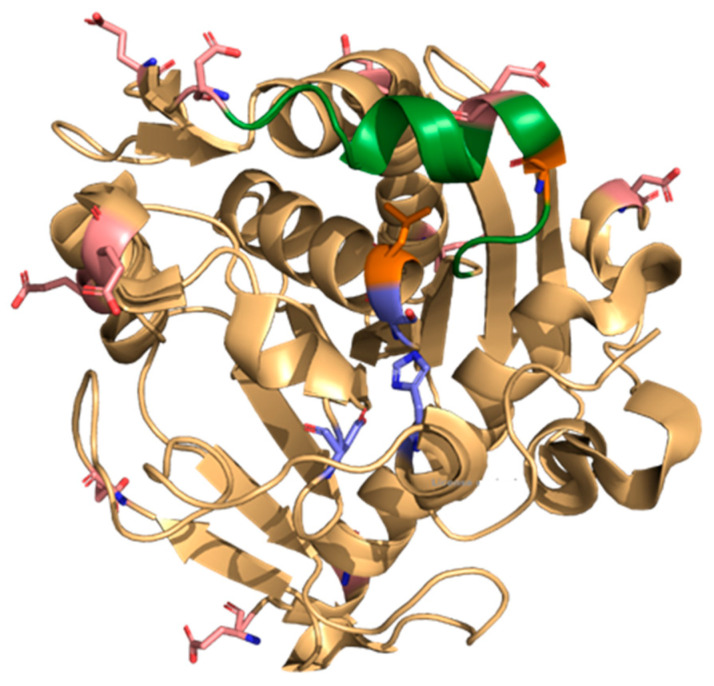
Structural model of TLL highlighting key regions related to catalytic function. The lid region (R81–F94) is shown in green, oxyanion hole (S85 and L147) is shown in orange, and catalytic residues S146, D201, and H258 in blue. Of the 32 possible carboxylate groups, 31 were found to be exposed to modification. The groups modified in silico, based on the average modification percentage for TLL, are highlighted in pink.

Root means square fluctuation (RMSF) analysis (Figure 3C) revealed localized flexibility increases in key catalytic regions: R81–I100 and V140–D165, including the lid (R91–D96), oxyanion hole (S83, L147), and catalytic serine (S146), mainly in hydrazide-modified variants (Figure 4). Particularly, longer-chain hydrazide modifiers induced lower lid mobility in the open form (Figure 2 and Figure S3), consistent with the RMSF profile (Figure 3C). Hence, the lid region (R91–D96) of TLL displayed progressive increases in conformational fluctuations as the modifier size decreased (AA: ~1.2 Å; SC: ~2.9 Å; OX: ~3.0 Å; CZ: ~4.7 Å). These results indicate that the lid remains relatively rigid in the AA-modified enzyme, whereas in the CZ-modified form it exhibits nearly fourfold greater fluctuations, correlating with the observed enhancement in catalytic efficiency. The longer, more flexible chains likely facilitate polar contacts through specific *H*-bond interactions (particularly between AA-hydrazide96 (AAH96) and R108, and between AAH96 and AAH99) that limit lid movement (Figure S7), thereby stabilizing the open, catalytically active conformation and facilitating substrate access. These enhanced fluctuations may correlate with improved substrate access and turnover, suggesting a mechanistic link between increased local flexibility and catalytic efficiency. This structural plasticity—induced by chemical modification—supports the adoption of more catalytically competent conformations compared with those adopted by unmodified versions, as previously suggested by Han et al. [39].

Previous work has demonstrated that replacing negatively charged residues (Asp, Glu) with neutral or basic ones (e.g., Phe, Lys) can disrupt salt bridges and increase flexibility, thereby enhancing catalytic performance [39,40,41]. In line with this, converting surface β- or γ-carboxylates into neutral hydrazide groups in our study may increase conformational flexibility and contribute to the observed improved activity of TLL variants CZ, SC and AA (Table 2).

The observed kinetic trends correlate with molecular docking data found (Table 3), where more efficient variants exhibited lower binding energy scores in their enzyme–substrate complexes with *p*-NPA, supporting improved substrate recognition or accessibility.

#### 2.2.3. Kinetic Studies of MTL and Its Variants

**Scheme 3:**
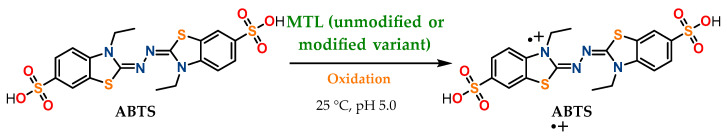
Oxidation of ABTS catalyzed by native and chemically modified MTL variants. The formation of the ABTS^•+^ radical cation (λ = 420 nm) was monitored spectrophotometrically, as described in Section 3.2.

The kinetic parameters for the unmodified MTL and its chemically modified variants reveal significant differences in catalytic behavior during the oxidation of ABTS at 25 °C and pH 5.0 (Table 4; Scheme 3, Supporting Materials Figure S14). As observed with TLL, the chemical modification of MTL also led to improved catalytic performance for most variants (Table 4), similar to what was found with TLL, without a clear correlation to the chain length of the dihydrazides. The unmodified enzyme displayed moderate catalytic parameters (k_cat_ = 6.5 s^−1^, k_m_ = 12.2 μM), while the variants MTL-OX and MTL-AA reached catalytic efficiencies of 2.1 μM^−1^ s^−1^—four times better than that of the unmodified enzyme. These improvements were primarily due to increased turnover (e.g., MTL-OX, k_cat_ = 22.7 s^−1^) and/or enhanced substrate affinity (e.g., MTL-EDA, k_m_ = 4.5 μM). In contrast to what is shown for TLL, MTL-CZ exhibited reduced turnover and comparable affinity, resulting in no significant improvement.

Docking results (Table 5) partially supported these trends. While most modified variants exhibited lower ligand-receptor interaction energies (more favorable binding), no direct correlation between docking scores and k_m_ values was observed. This discrepancy may arise from the presence of an oxidized histidine (O-His98) in the crystallographic structure used (PDB:6F5K), which can alter the binding cavity conformation and substrate accessibility (Supporting Materials Figures S15–S20), as previously reported [42,43].

**Figure 5:**
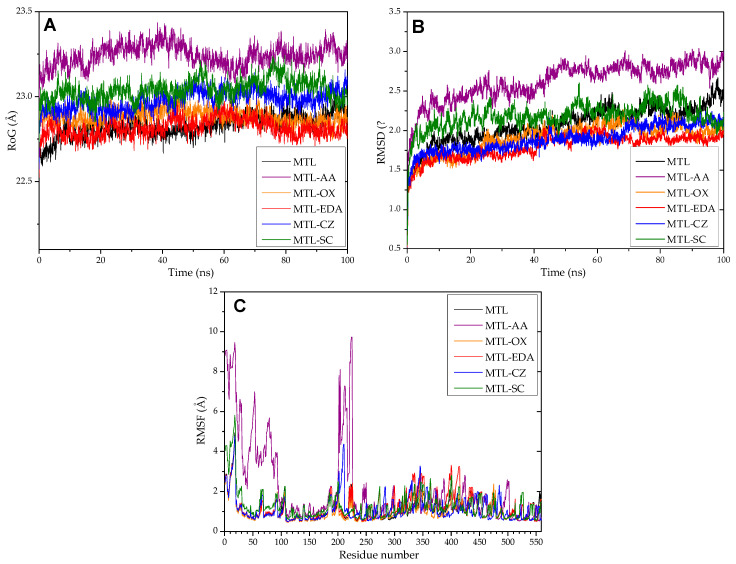
(**A**) radius of gyration (RoG), (**B**) Root mean square deviation (RMSD), and (**C**) root mean square fluctuation (RMSF) plots of the MTL and modified versions produced after 100-ns molecular dynamics simulations. MTL (black line, PDB:6F5K); MTL-AA (purple line); MTL-OX (orange line); MTL-EDA (red line); MTL-CZ (blue line), and MTL-SC (green line).

The MD simulations for MTL (Figure 5) revealed structural behaviors analogous to TLL (Section 2.2.2). All MTL-modified variants exhibited initial equilibration followed by stabilization in their RMSD profiles, suggesting that a dynamic conformational equilibrium was reached. The unmodified MTL (black line) maintained the lowest and most stable RoG (~22.7 Å), indicating a compact and consistent structure, similar to MTL-EDA (red line) and MTL-OX (orange line), which showed only slight increases (~22.7–22.9 Å), suggesting minor conformational changes. In contrast, MTL-AA (purple line) exhibited the highest RoG values (~23.1–23.4 Å), indicating structural expansion likely due to increased surface flexibility or disrupted internal packing. MTL-SC (green) and MTL-CZ (blue) also displayed elevated RoG values (~23.0–23.2 Å), representing moderate relaxation. Interestingly, as also observed for TLL, the effect tended to increase with longer chain modifiers, with EDA and OX (comparable in length) showing similar RoG values (Figure S6, Supporting Materials). In contrast, CZ again deviated from this trend, producing disproportionately higher conformational changes not seemingly explained by steric effects alone.

Consistent with this, RMSD (Figure 5B) trajectories showed that all systems initially equilibrated during the first 20–30 ns before stabilizing. MTL-AA showed the highest structural fluctuations, with RMSD values > 2.7 Å, indicating significant conformational changes. In comparison, MTL-CZ, MTL-EDA, and MTL-OX maintained low RMSD values (~2.0–2.1 Å), which reflect improved structural stability, while the unmodified enzyme remained within a moderate range (~2.0–2.5 Å). MTL-SC demonstrated intermediate behavior, characterized by a gradual increase followed by a plateau, indicating greater flexibility without compromising overall integrity. These results suggest that while structural relaxation can enhance catalytic dynamics, excessive expansion—such as in MTL-AA—must be carefully balanced to prevent destabilization, as discussed in Section 2.3.

**Figure 6:**
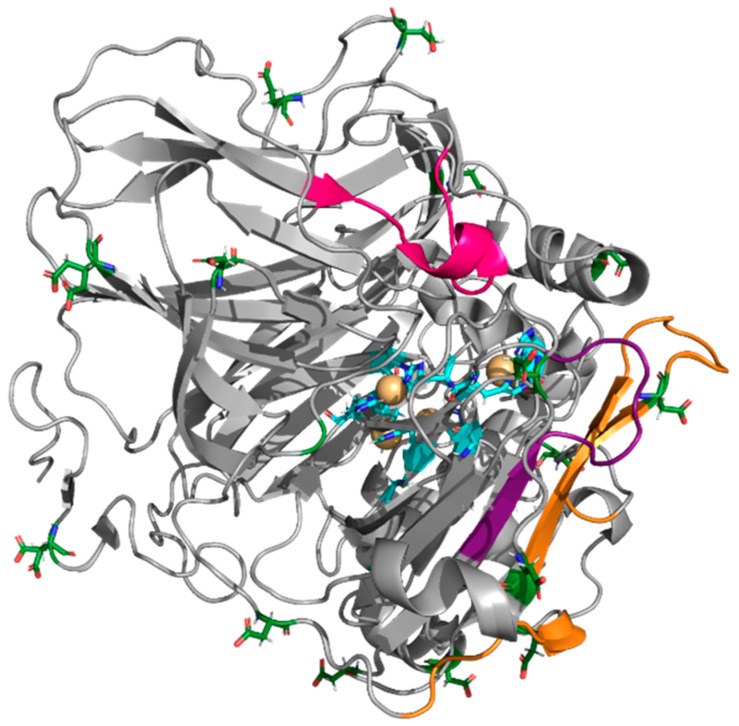
Structural model highlighting regions with notable conformational shifts observed exclusively in MTL-OX (G350–N376 in orange) and MTL-AA (V290–S301 in pink, G350–P401, L418–T428 in purple). These regions are located near the T1 copper site and may influence the catalytic performance. Of the 65 possible carboxylate groups, only 42 were found to be modifiable. The groups modified in silico, based on the average modification percentage for MTL, are highlighted in green.

The RMSF analysis (Figure 5C) further showed localized increases in mobility across all variants. The most notable fluctuations occurred in distal loops; however, significant conformational shifts near the T1 copper center were exclusive to MTL-OX (G350–N376) and MTL-AA (V290–S301, G350–P401, and L418–T428) (Figure 6). These flexible regions likely modulate substrate access or redox activity and may explain the enhanced catalytic turnover observed for these variants [6,42].

In this sense, as was shown with TLL, chemical modifications induced overall and localized flexibility changes in MTL, thereby affecting substrate binding and turnover. These findings align with the study by Pinheiro et al. [6], which demonstrated that amination enhances MTL activity by inducing conformational changes, including a reduction in α-helices. However, at higher modification levels, this effect can become detrimental due to excessive structural disruption [6,44,45,46,47,48]. The above could explain why the EDA-modified enzyme in the present study showed a lower k_cat_ compared to the unmodified enzyme, especially considering that the applied methodology yielded a 44.6 ± 1.9% of modification. In contrast, despite a similar degree of modification observed for OX, SC, and AA, these variants exhibited higher catalytic turnover. This suggests that the structural and functional responses depend not only on the extent of modification but also on the physicochemical properties of the modifiers employed.

It should also be noted that EDC-mediated activation can target not only carboxyl groups for hydrazide or amine coupling but may also react with cysteine and tyrosine residues [6]. These additional reactions, in combination with the intended modification, may contribute to the effects observed in the modified enzymes (TLL or MTL) compared to the unmodified ones. Nevertheless, since all enzymes underwent the same activation protocol, the differential responses can be attributed primarily to the nature of the hydrazide used.

### 2.3. Thermal and Organic Solvent Stability of Modified Enzymes

**Figure 7:**
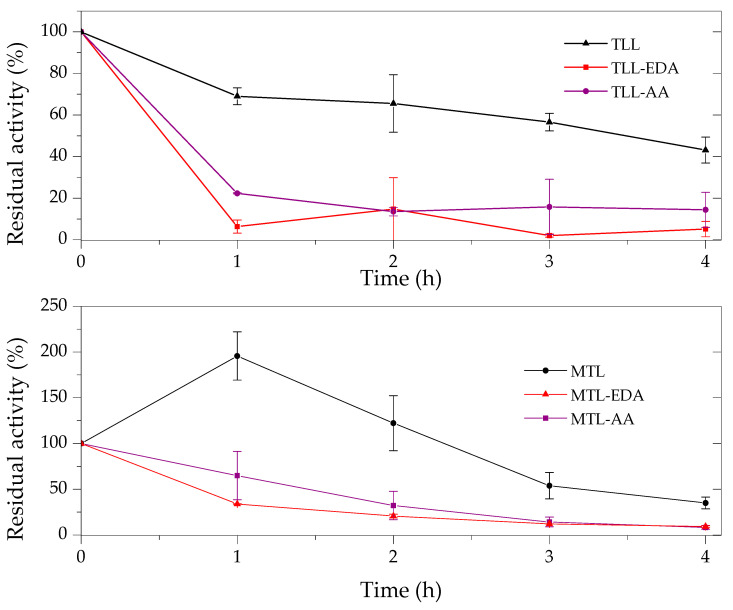
Residual activity (%) over time at 70 °C for TLL (top: native [black], TLL-EDA [red], TLL-AA [purple]) and MTL (bottom: MTL-EDA [red], MTL-AA [purple]). Residual activity was calculated as 100 × ([X]_s_/[X]_0_), where [X]_s_ is activity at time t and [X]_0_ is initial activity (t = 0 h).

Enzyme functionality under harsh conditions, such as high temperatures or the presence of organic solvents, is crucial for industrial applications. Based on their consistent superior catalytic performance (Section 2.2), AA-modified enzymes (TLL-AA and MTL-AA) were selected for thermal and solvent stability assays at 70 °C and in 50% (v/v) THF (25 °C), which is an emerging low-toxicity solvent relevant to biomass processing [49]. For comparison, unmodified and EDA-modified versions were also evaluated (Figure 7).

At 70 °C, both modified versions of TLL (EDA and AA) exhibited a faster decline in activity compared to the unmodified enzyme. This agrees with MD simulations at 70 °C (Supporting Materials, Figure S21, A.I and B.I), where both modified forms displayed higher RoG values and RMSD profiles with greater fluctuations, indicating increased conformational expansion and reduced compactness. Nevertheless, their trajectories plateaued after ~50 ns (Figure S21, B.I), implying that they eventually adopted energetically stable, albeit less or not active (Figure 7, top), conformations. Complementarily, RMSF profiles further suggested that thermal-induced flexibility was distributed across the protein structure, with each variant showing a distinct conformational structure (Supporting Materials, Figure S21, C.I).

For unmodified MTL at 70 °C, two-step behavior was observed: a transient rise in activity followed by progressive deactivation (Figure 7, bottom). MD simulations support this finding, showing an initial increase in flexibility (RoG and RMSD, Supporting Materials, Figure S21, A.II and B.II) without stabilization over the simulation timeframe. These findings indicate that the initial activity rise may stem from thermally induced flexibility that briefly enhances substrate access; however, prolonged exposure likely causes globularity loss and less active conformations. In contrast, MTL-AA and MTL-EDA reached structural stabilization earlier, mirroring TLL behavior. However, as with TLL, that stabilization did not translate to higher residual activity, highlighting that while chemical modifications can accelerate transitions toward energetically favorable states, these states are not catalytically unfavorable. Altogether, these findings underscore the trade-off between flexibility and stability under thermal stress and reinforce the need to balance both aspects when designing robust biocatalysts [39,40,41,44,45,46,47,48].

**Figure 8:**
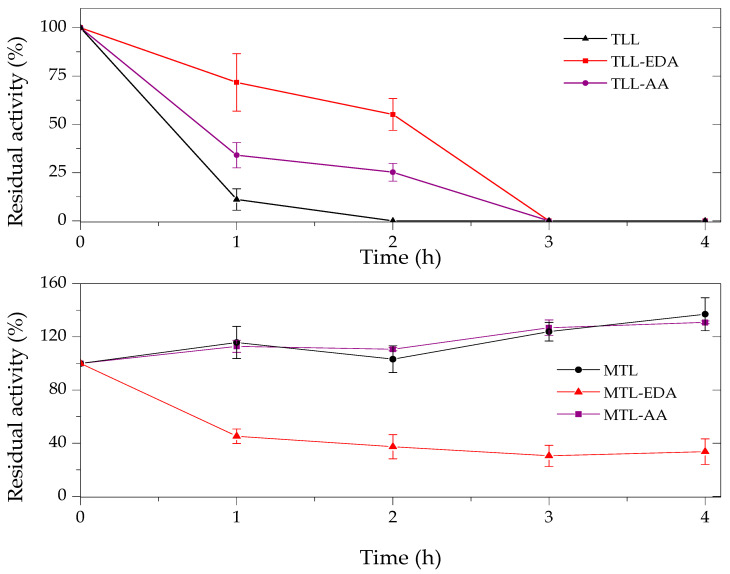
Residual activity over time for TLL (top) and MTL (bottom) variants—unmodified (black), EDA-modified (red), and AA-modified (purple)—in 50% (v/v) THF at 25 °C. Residual activity was calculated as 100% × ([X]_s_/[X]_0_).

In the presence of solvent (50% THF (v/v) at 25 °C), the chemically modified enzymes exhibited distinct behaviors in comparison to their native forms. For TLL (Figure 8, top), the EDA-modified variant retained activity for longer periods than both the unmodified and AA-modified forms. This improved stability aligns with MD simulations (Supporting Materials, Figure S22), where TLL-EDA displayed higher RoG and RMSD values than the native enzyme, indicative of greater conformational mobility. However, unlike the thermal simulations, the native TLL rapidly adopted a stable structure in THF, but this conformation seems catalytically inactive, suggesting that structural stability alone does not guarantee functional persistence. In contrast, the conformational plasticity of TLL-EDA may facilitate the maintenance of active conformers under solvent stress, thus explaining its superior performance.

In the MTL case (Figure 8, bottom), the EDA-modified form showed a marked loss of stability, while both the native enzyme and MTL-AA maintained comparable activity levels throughout the incubation period. This loss in MTL-EDA may be due to high amination levels, previously associated with decreased laccase stability [6,45,47]. By contrast, neutral or negatively charged groups have been reported to maintain or even enhance stability [44,46,48], consistent with the observed stability of MTL-AA. MD simulations in THF for MTL showed structural differences among the variants (Supporting Materials, Figure S22). Notably, the P320–L370 region in MTL-EDA showed increased flexibility, as indicated by RMSF data, which could disrupt the nearby copper catalytic. Meanwhile, in MTL-AA, the F200–R226 segment—remote from the active site—exhibited significant flexibility under both thermal and solvent stress, indicating that this region may function as a structural modulator, aiding in the accessibility or orientation of catalytic domains (Supporting Materials, Figure S22, C.II).

### 2.4. Preliminary Assessment of Biotechnological Applications Using Enzyme Variants

The use of enzyme-based technologies, as opposed to conventional chemical processes, is often associated with reduced greenhouse gas emissions, lower energy consumption, and, in some cases, reduced operational costs. For example, industrial biodiesel production using lipases has been reported to decrease these parameters by up to 37%, 79%, and 87%, respectively [50]. Moreover, laccases have been linked to progress toward net-zero carbon emission targets, as they enable more efficient biomass valorization and bioremediation than conventional chemical approaches [51]. Accordingly, two model reactions were employed to assess the biotechnological potential of the modified enzyme variants.

In a one-step, solvent-free transesterification assay for biodiesel synthesis [38], neither unmodified nor modified TLL (EDA or AA) produced detectable ethyl esters, confirming their sensitivity to ethanol-induced inactivation [38,52] (Supporting Materials, Table S5). Thus, the chemical modifications applied here did not prevent enzyme deactivation under these conditions, aligning with the results found in solvent stability for TLL.

In contrast, enzymatic dye decolorization assays using 25 ppm indigo carmine revealed modest improvements for modified MTL. After 6 h, MTL-EDA and MTL-AA achieved bleaching yields of 24.1 ± 2.5% and 22.8 ± 2.8%, respectively, compared to 16.0 ± 1.8% for the unmodified enzyme (Figure S23). Docking analyses (Supporting Materials, Figures S24–S26) supported these experimental observations by indicating comparable or slightly improved substrate accommodation for the modified variants.

Achieving industrial viability for enzymes is most likely when chemical modification is combined with complementary stabilization strategies. As demonstrated, our approach—tailoring the size of the modifying agent (e.g., employing dihydrazides instead of ethylenediamine)—does not inherently confer enhanced stability. Rather, it should be regarded as a versatile tool primarily for improving catalytic activity, which can be further potentiated when integrated with stabilization techniques [3,10,15].

## 3. Materials and Methods

### 3.1. Materials

*Thermomyces lanuginosus* lipase, *Myceliophthora thermophila* laccase, Q-Sepharose^®^, *p*-nitrophenyl acetate (*p*-NPA), 2,2′-azino-bis(3-ethylbenzothiazoline-6-sulfonic acid) (ABTS) Cetyltrimethylammonium bromide (CTAB), sodium dodecyl sulfate, ethylenediaminetetraacetic acid (EDTA), bicinchoninic Acid Kit (BCA), bovine serum albumin (BSA), ethanol (96%), 1-ethyl-3-(3-dimethylaminopropyl)carbodiimide (EDC), ethylenediamine, carbohydrazide, oxalyl dihydrazide, succinyl dihydrazide, adipic acid dihydrazide, Sephadex G-100, Sephadex G-50, and salts for buffering solutions were purchased from Sigma Chem. Co. (St. Louis, MO, USA). PEI-agarose support was prepared according to Guisán [53]. Palm olein was purchased from a local market. Unrefined palm oil and used cooking oil were generously donated by Biocombustibles Sostenibles del Caribe S.A. (Santa Marta, Colombia). All other reagents and solvents were of analytical or HPLC grade. The Lewatit^®^ VP OC 1600 (LW) support, based on polymethacrylate/divinylbenzene copolymer, was kindly donated by Lanxess^®^ (Cologne, Germany).

### 3.2. Esterase and Oxidative Activity Determination

Esterase activity of TLL toward *p*-nitrophenyl acetate hydrolysis (*p*-NPA, 0.4 mM) was determined at pH 7.0 (25 mM sodium phosphate buffer, 25 °C) as previously described [29,38], with the addition of 0.001% CTAB and 0.01% Triton X-100. When testing variable surfactant concentrations, these were adjusted accordingly. The oxidative activity of MTL was measured via ABTS oxidation (180 µM) at pH 5.0 (40 mM sodium acetate buffer, 25 °C), following standard procedures [6,54]. The formation of ABTS^•+^ was monitored at 420 nm (ε_420_ = 3.6 × 10^4^ M^−1^ cm^−1^). One international unit (IU) corresponds to the amount of enzyme that converts 1 μmol of substrate per minute under the specified conditions.

### 3.3. Protein Determination

Protein content was measured using either the Bradford reagent or the Pierce^®^ BCA (manufactured by Thermo Fisher Scientific, Waltham, MA, USA), with BSA as the standard protein [55].

### 3.4. Solid-Phase Enzyme Modification

Solid-phase modifications were performed following established procedures [3,21]. TLL was immobilized on Lewatit^®^ VPOC1600 or Q-Sepharose^®^ and MTL were immobilized on the last one support or PEI-agarose under mild conditions (pH 7.0, 35 °C) until ≥99% of the activity was bound, yielding derivatives with 32 mg/g (TLL) and 14 mg/g (MTL). The immobilized enzymes were subsequently modified with 0.5 M ethylenediamine (EDA) or adipic acid dihydrazide (AA) in the presence of 10 mM EDC at pH 4.75 and 35 °C for 3 h. Reactions were stopped by adjusting the pH to 7.0. Desorption was carried out with buffered solutions containing surfactants (CTAB for TLL, Triton X-100 and NaCl for MTL) as previously described [3,53].

### 3.5. Liquid-Phase Enzyme Modification

Soluble enzymes (MTL or TLL, 4 mg/mL) were modified in the liquid phase using the modifiers EDA, carbohydrazide (CZ), oxalyl dihydrazide (OX), succinyl dihydrazide (SC), and AA, with 10–70 mM EDC and 0.5 M modifier, following the same procedure described in Section 3.4. The modified enzymes were purified as described in Section 3.6.

### 3.6. Purification of Modified Enzymes

Excess modifiers were removed by size-exclusion chromatography using Sephadex^®^ G-50 (TLL) or G-100 (MTL), as described previously [56]. Protein recovery was assessed by the Bradford assay (Section 3.3), and active fractions were concentrated using centrifugal ultrafiltration tubes (10 kDa cutoff for TLL, 50 kDa for MTL). Final enzyme solutions were stored at 4 °C. Unmodified enzymes were processed in parallel as controls.

### 3.7. Determination of Enzyme Surface Modification

The yield of surface modification was determined using the 2,4,6-trinitrobenzene sulfonic acid (TNBS) assay, which quantifies free amines or hydrazides [57,58]. Enzyme samples in sodium borate buffer (pH 8.5) were incubated with 0.008% (w/v) TNBS at 30 °C and 800 rpm for 40 min. The reaction was quenched with a sodium carbonate buffer (pH 10.8), and the absorbance was measured at 420 nm for EDA derivatives and 500 nm for hydrazide-modified enzymes. Standard curves were generated using BSA (lysine equivalents) and octanoic hydrazide. The extent of modification was calculated by comparing modified and unmodified samples and normalized to the number of accessible carboxyl groups (32 for TLL, 42 for MTL). Full details and the calculation formula are available in the Supporting Information (Table S6, Figures S27 and S28 and Equation (S1)).

### 3.8. SDS-PAGE Analysis

SDS-PAGE was conducted using 5% stacking and 12% resolving polyacrylamide gels. Samples (0.25–0.3 mg/mL) were prepared with 8% SDS and 10% β-mercaptoethanol and denatured by boiling. Protein bands were visualized using Coomassie Brilliant Blue. Molecular weights were estimated using a Bio-Rad LMW-SDS marker (10–250 kDa) [59]. Densitometric analysis was performed using ImageJ software (version 1.54d) as previously described [60]. Full analysis details are provided in the Supporting Information.

### 3.9. Michaelis–Menten Kinetics

Kinetic parameters were determined using 1 ng/mL enzyme and substrate concentrations: 0–4 mM *p*-NPA (TLL) [61,62] or 0–180 μM ABTS (MTL) [63,64], as described in Section 3.2. The resulting Michaelis–Menten curves were fitted using OriginPro 9.0 and the Levenberg–Marquardt algorithm [65]. The k_cat_ values (derived from Vmax) were calculated assuming that all measured protein content corresponded to the target active enzyme after performing the procedure described in Section 3.6.

### 3.10. Solvent and Thermal Stability Assays

Residual enzyme activity was expressed as the percentage of the initial activity (t_0_) remaining over time under deactivating conditions [66,67]. The inactivation course of the enzymes at 70 °C in 50 mM sodium phosphate (pH 7.0) or 50:50 (v/v) THF:H_2_O (pH 7.0 and 25 °C) was conducted as previously described [66,67].

### 3.11. Preliminary Assessment of the Obtained Enzymes in Biotechnological Applications

#### 3.11.1. One-Step Solvent-Free Fatty Acid Ethyl Ester Synthesis (FAEs)

A one-step transesterification reaction was performed by adding lipase (1.3 mg) to palm olein (0.7 g) and absolute ethanol (~120 mg), using a 3.1:1 ethanol: oil molar ratio, without solvents or additives. The yield was analyzed using FT-IR with a PCA method, following previously reported methods [29,38].

#### 3.11.2. Indigo Carmine Decolorization

For dye decolorization, laccase (1.2 mg) was added to 1 mL of a 25 ppm indigo carmine solution in a 2 mL vial. The mixture was incubated at 25 °C and 170 rpm for the desired time. Decolorization rate was assessed by measuring the absorbance decrease at 611 nm, and expressed as a decolorization percentage relative to the control with the enzyme inhibited with sodium azide [68].

### 3.12. Molecular Dynamics and Molecular Docking Studies of Modified Enzymes

Crystal structures of *T. lanuginosus* lipase (TLL, PDB:1DTE) and *M. thermophila* laccase (MTL, PDB:6F5K) were retrieved from the Protein Data Bank [42,69]. Residues were selected for in silico modification based on experimental modification percentages, applying dihydrazide moieties proportionally to the exposed residues, and distributed homogeneously across the enzyme surface using PyMOL v.4.6.0 and AVOGADRO2 v1.97.0. Structures were exported in .pdb format and refined through 2-ns molecular dynamics (MD) simulations in YASARA Structure v.19.12.14, using the AMBER14 force field and the Berendsen thermostat and manometer barostat [70,71]. Molecular dynamics (MD) simulations were carried out on a 48-core server using periodic boundary conditions. The protein–ligand complex was positioned at the center of a cubic box (20 Å beyond the protein surface), solvated with the simple point charge (SPC) water model, and neutralized by randomly replacing water molecules with Na^+^ and Cl^−^ ions to achieve a 0.9% NaCl concentration. System relaxation was performed in multiple stages: initially, the protein was fixed, and solvent molecules were optimized by steepest descent and local steepest descent algorithms to remove steric clashes. The protein backbone was then restrained, and the solvent was further relaxed with the same procedure. After releasing restraints, a short 10-ps dynamics run was performed to equilibrate the system. Long-range van der Waals interactions were treated using a spherical cut-off of 0.8 nm. Production simulations were then performed under NPT conditions (1 bar, 298 K), pH 7.4, and a density of 0.997 g/mL, with a time step of 2.5 fs and snapshots recorded every 100 ps. The best-refined structure (quality score ≥ 0.3) was selected and simulated for 100 ps [72]. To assess environmental effects, additional simulations were conducted at elevated temperature (343 K) and in a mixed-solvent system containing 50% THF. Trajectory visualization and analyses were performed using YASARA and PyMOL.

## 4. Conclusions

Eight novel biocatalysts were generated by chemically modifying two distinct enzymes, TLL and MTL, with dihydrazides (CZ, OX, SC, AA), using a liquid-phase methodology adapted for this study. The modified enzymes were compared with their EDA-modified counterparts. Notably, the hydrazide-based protocol yielded modification percentages comparable to amination under the same aqueous-phase methodology, while offering easier pH adjustment and easier purification, regardless of the length of the dihydrazide used. Additionally, no perceptible vapors were generated during handling, unlike EDA-modifications, an important safety and environmental advantage despite the higher reagent cost. This aqueous-phase approach proved to be widely applicable to two different types of enzymes and enabled the generation of variants with significantly improved catalytic efficiency (up to 2.5 times for TLL-CZ and 4.2 times for MTL-OX and MTL-AA), improving the surfactant activation effect, outperforming their unmodified and EDA-modified counterparts. AA-modification was consistently the best-performing across both enzymes in catalytic terms, whereas CZ and OX conferred benefits specific to TLL of MTL, respectively.

Molecular dynamics simulations revealed that the catalytic improvements observed after chemical modification were associated with moderate increases in RoG and region-specific RMSF increments near the catalytic sites, reflecting greater local flexibility, likely arising from the disruption of stabilizing interactions. Furthermore, docking analyses yielded complementary insights by predicting variations in substrate-binding modes that were consistent with the experimentally determined catalytic efficiencies. These simulations provided mechanistic support and structural insights, linking dihydrazide-induced flexibility to improved catalysis and highlighting their potential for rational enzyme functionalization.

Despite these advances, the modified enzymes showed no improvement in preliminary applications such as biodiesel synthesis or dye degradation, indicating that catalytic enhancement with model substrates under standard conditions does not necessarily translate to improved performance under real operational conditions. Therefore, to unlock its full biotechnological potential, future strategies should explore the integration of chemical modification with enzyme immobilization or other stabilization approaches that preserve activity while improving operational performance.

## Figures and Tables

**Table 1:** Summary of TLL and MTL modifications with EDA and dihydrazides (CZ, OX, SC, AA), including modification percentage, protein and activity recovery, and specific activity (IU/mg).

Enzyme	Modifier	Abbr.	Modification (%)	Recover Protein (%)	Recover Activity (%)	Specific Activity (IU/mg)
*Thermomyces lanuginosus* lipase (TLL)	-	TLL ^a^	-	87.3 ± 0.2	90.0 ± 0.4	9.4 ± 0.2
	Ethylenediamine (EDA)	TLL-EDA	47.1 ± 0.2	48.4 ± 1.0	29.4 ±3.4	5.9 ± 0.5
	Carbohydrazide (CZ)	TLL-CZ	57.1 ± 1.2	85.0 ± 1.4	128.6 ± 5.7	14.7 ± 0.4
	Oxalyl hydrazide (OX)	TLL-OX	53.2 ± 3.6	68.5 ± 2.1	2.1 ± 0.3	0.3 ± 0.1
	Succinic dihydrazide (SC)	TLL-SC	45.7 ± 0.9	98.1 ± 3.1	123.5 ± 2.8	12.2 ± 0.3
	Adipic acid dihydrazide (AA)	TLL-AA	66.0 ± 0.3	83.7 ± 1.7	159.8 ± 3.8	18.6 ± 0.6
*Myceliophthora thermophila* laccase (MTL)	-	MTL ^a^	-	69.4 ± 0.4	95.2 ± 4.9	1.5 ± 0.3
	Ethylenediamine (EDA)	MTL-EDA	44.6 ± 1.9	66.4 ± 0.6	186.3 ± 6.3	4.4 ± 0.2
	Carbohydrazide (CZ)	MTL-CZ	16.2 ± 0.8	95.1 ± 1.5	23.2 ± 2.8	0.4 ± 0.1
	Oxalyl hydrazide (OX)	MTL-OX	54.2 ± 3.1	85.7 ± 0.2	122.4 ± 5.7	2.4 ± 0.2
	Succinic dihydrazide (SC)	MTL-SC	41.9 ± 0.7	64.6 ± 0.4	231 ± 6.1	5.6 ± 0.2
	Adipic acid dihydrazide (AA)	MTL-AA	40.6 ± 1.2	71.0 ± 1.0	389 ± 2.9	8.6 ± 0.2

^a^ Control of the unmodified enzyme after purification. The activity and protein content were measured using the procedures described in Section 3.2 and Section 3.3. The activity and recovered protein were calculated using a modification target [X]_0_ and the values obtained after modification and desorption [X]_m_, using the equation 100% ([X]_m_/[X]_0_). The modification percentage was calculated using TNBS method, as described in Section 3.7.

**Table 2:** Kinetic parameters of unmodified and modified TLL in the hydrolysis of *p*-NPA (25 °C, pH 7.0, CTAB 0.01%) (Scheme 2). Reported values include turnover frequency (k_cat_), Michaelis–Menten constant (k_m_), and catalytic efficiency (k_cat_/k_m_).

Enzyme	k_cat_ (s^−1^)	k_m_ (mM)	k_cat_/k_m_ (mM^−1^ s^−1^)
TLL	70.1 ± 7.5	1.3 ± 0.2	55.6 ± 8.2
TLL-EDA	42.4 ± 2.6	1.0 ± 0.2	44.6 ± 3.2
TLL-CZ	98.0 ± 8.4	0.7 ± 0.3	138.0 ± 9.1
TLL-OX	8.9 ± 0.9	1.2 ± 0.2	7.3 ± 1.7
TLL-SC	100.4 ± 9.4	1.1 ± 0.2	90.5 ± 7.1
TLL-AA	78.0 ± 5.2	1.0 ± 0.1	75.0 ± 6.3

**Table 3:** Docking scores (kJ/mol) of TLL and modified variants using the complex with CTAB as a receptor [29] and *p*-NPA as substrate (see Supporting Materials Figures S8–S13).

Enzyme	Docking Scores (kJ/mol)—Enzyme with CTAB	Standard Deviation (SD)
TLL	−60.1	1.0
TLL-EDA	−51.8	0.2
TLL-CZ	−76.9	1.0
TLL-OX	−76.9	0.7
TLL-SC	−71.0	0.5
TLL-AA	−67.7	0.8

**Table 4:** Kinetic parameters of MTL and MTL modified in the oxidation of ABTS (25 °C, pH 5.0) (Scheme 3). Reported values include turnover frequency (k_cat_), Michaelis–Menten constant (k_m_), and catalytic efficiency (k_cat_/k_m_).

Enzyme	k_cat_ (s^−1^)	k_m_ (μM)	k_cat_/k_m_ (μM^−1^ s^−1^)
MTL	6.5 ± 0.5	12.2 ± 0.3	0.5 ± 0.2
MTL-EDA	6.1 ± 0.3	4.5 ± 0.2	1.4 ± 0.1
MTL-CZ	4.1 ± 1.1	8.7 ± 0.2	0.5 ± 0.2
MTL-OX	22.7 ± 9.1	11.1 ± 0.7	2.1 ± 0.8
MTL-SC	6.7 ± 0.6	8.5 ± 0.4	0.8 ± 0.2
MTL-AA	13.1 ± 0.4	6.2 ± 0.4	2.1 ± 0.2

**Table 5:** Summary of docking result of MTL and modified as a receptor and ABTS as ligand (see Supporting Materials Figures S15–S20).

Enzyme	Score Ligand-Receptor (kJ/mol)	Standard Deviation (SD)
MTL	−110.6	1.0
MTL-EDA	−141.3	5.4
MTL-CZ	−103.0	0.6
MTL-OX	−120.6	0.9
MTL-SC	−132.6	0.4
MTL-AA	−173.5	2.2

## Data Availability

The original contributions presented in this study are included in the article/Supplementary Materials. Further inquiries can be directed to the corresponding author(s).

## Supplementary Materials

The supporting information can be downloaded at: https://www.mdpi.com/article/10.3390/ijms262211094/s1.

## Abbreviations

The following abbreviations are used in this manuscript:

AA	Adipic acid dihydrazide
ABTS	2,2′-Azino-bis(3-ethylbenzothiazoline-6-sulfonic acid)
CZ	Carbohydrazide
EDA	Ethylenediamine
MD	Molecular dynamics
MTL	*Myceliophthora thermophila* laccase
OX	Oxalyl dihydrazide
PDB	Protein Data Bank
RoG	Radius of gyration
RMSD	Root mean square deviation
RMSF	Root mean square fluctuation
SC	Succinyl dihydrazide
T1	Type 1 copper center
THF	Tetrahydrofuran
TLL	*Thermomyces lanuginosus* lipase
