# Supplementary material for: Chemical Modification of Thermomyces lanuginosus Lipase and Myceliophthora thermophila Laccase Using Dihydrazides: Biochemical Characterization and In Silico Studies
Source: Int J Mol Sci. 2025 Nov 16;26(22):11094. doi: 10.3390/ijms262211094 (PMC12652127; doi:10.3390/ijms262211094)
Supplement: Supplementary file 1 [file ijms-26-11094-s001.zip › ijms-3945995-supplementary.pdf]

Supplementary Material

Chemical Modification of *Thermomyces lanuginosus* Lipase and *Myceliophthora thermophila* Laccase Using Dihydrazides: Biochemical Characterization and In Silico Studies

Juan S. Pardo-Tamayo [1,*], Maria Camila Muñoz-Vega [1], Oscar L. Alférez [1], Evelyn L. Guerrero-Tobar [1], Chonny Herrera-Acevedo [2], Ericsson Coy-Barrera [3] and César A. Godoy [1,*]

[1] Laboratorio de Investigación en Biocatálisis y Biotransformaciones (LIBB), Grupo de Investigación en Ingeniería de los Procesos Agroalimentarios y Biotecnológicos (GIPAB), Department of Chemistry , Universidad del Valle, Cali 760001, Colombia; moreno.oscar@correounivalle.edu.co (O.L.A.); evelyn.guerrero@correounivalle.edu.co (E.L.G.-T.)

[2] Department of Chemical Engineering, Universidad ECCI, Bogotá 111311, Colombia; cherreraa@ecci.edu.co

[3] Bioorganic Chemistry Laboratory, Facultad de Ciencias Básicas y Aplicadas, Universidad Militar Nueva Granada, Cajicá 250247, Colombia; ericsson.coy@unimilitar.edu.co

* Correspondence: juan.sebastian.pardo@correounivalle.edu.co (J.S.P.-T.); cesar.godoy@correounivalle.edu.co (C.A.G.)

Table of Contents

1. Preliminary results of enzyme modification 3

2. SDS-PAGE gels analysis 4

3. Lid dynamics study (TLL) 6

4. Characterization of modified versions of TLL and MTL 7

5. Thermal and organic solvent stability of different enzyme variants 15

6. Preliminary assessment of biotechnological applications using enzyme variants 17

7. Experimental procedures details 19

7.1 Determination of percentage of modification using TNBS assay. 19

7.2 SDS-PAGE densitometry analysis 21

1. Preliminary results of enzyme modification

Table S1. Modifications of TLL and MTL in solid phase in terms of percentage of activity and protein recovered.

Enzyme	Modifier	Support used	Recovered protein (%)	Recovered activity (%)
Thermomyces lanuginosus lipase (TLL)	EDA	Lewatit® VPOC1600	23.1 ± 1.6	16.3 ± 2.4
		Q-Sepharose	18.3 ± 3.4	4.1 ± 5.8
	AA	Lewatit® VPOC1600	19.2 ± 0.9	20.2 ± 1.1
		Q-Sepharose	17.8 ± 3.8	7.5 ± 4.6
Myceliophthora thermophila laccase (*MT*L)	EDA	PEI-agarose	0.5 ± 0.3	0.2 ± 0.4
		Q-Sepharose	11.7 ± 1.3	5.9 ± 2.6
	AA	PEI-agarose	0.1 ± 0.1	0.2 ± 0.1
		Q-Sepharose	8.3 ± 1.5	2.6 ± 1.9

The activity and protein content were measured using the procedure described in Sections 3.2 and 3.3. Activity and recovered protein were calculated based on the initial amount of derivative used for modification ($[X]_0$) and the values obtained after modification and desorption ($[X]_m$), using the equation: $100\% \times ([X]_m / [X]_0)$. For lipase, derivatives at 32 mg/g of enzyme were used, and for laccase, 14 mg/g, relative to the support.

Table S2. Liquid-phase lipase modification of TLL using 0.5 M of modifier with a concentration of protein of 4 mg/mL, varying the concentration of EDC.

Enzyme	Modifier	EDC (mM)	Protein recovered (%)	Activity recovered (%)	Modification (%)
Thermomyces lanuginosus lipase (TLL)	Control	-	75.3 ± 2.2	72.7 ± 4.1	-
	EDA	10	35.7 ± 1.1	17.8 ± 3.5	15.8 ± 2.3
		35	38.3 ± 2.1	21.0 ± 1.7	24.6 ± 1.4
		70	42.7 ± 1.7	23.4 ± 3.6	38.6 ± 0.7
	AA	10	73.6 ± 1.8	22.4 ± 1.1	16.4 ± 1.5
		35	68.9 ± 2.5	21.9 ± 0.8	23.0 ± 2.0
		70	72.2 ± 1.2	12.4 ± 0.2	40.2 ± 0.3

The activity and protein content were measured using the procedures described in Sections 3.2 and 3.3. The activity and recovered protein were calculated based on the initial modification target ($[X]_0$) and the values obtained after modification and desorption ($[X]_m$), using the equation: $100\% \times ([X]_m / [X]_0)$. The modification percentage was determined using the TNBS method, as described in Section 3.7.

Table S3. CTAB concentration effect in liquid-phase lipase modification.

Enzyme	Modifier	CTAB (% w/v)	Protein recovered (%)	Activity recovered (%)	Modification (%)
Thermomyces lanuginosus lipase (TLL)	EDA	0.01	43.1 ± 0.9	27.9 ± 4.1	22.0 ± 0.6
		0.10	48.4 ± 1.0	29.4 ± 3.4	47.1 ± 0.2
		1.00	52.3 ± 2.1	34.4 ± 5.8	49.6 ± 0.4
	AA	0.01	79.8 ± 4.3	59.8 ± 2.7	31.2 ± 0.7
		0.10	83.4 ± 1.7	159.8 ± 3.8	66.0 ± 0.3
		1.00	85.0 ± 2.3	143.6 ± 5.6	51.3 ± 0.5

Here, 28 mg of protein with an activity of 9 IU/mg was used. Activity and protein contents were measured using the procedures described in Sections 3.2 and 3.3. Activity and recovered protein were calculated based on the initial modification target ($[X]_0$) and the values obtained after modification and desorption ($[X]_m$), using the equation: $100\% \times ([X]_m / [X]_0)$. The modification percentage was determined using the TNBS method, as described in Section 3.7.

2. SDS-PAGE gels analysis

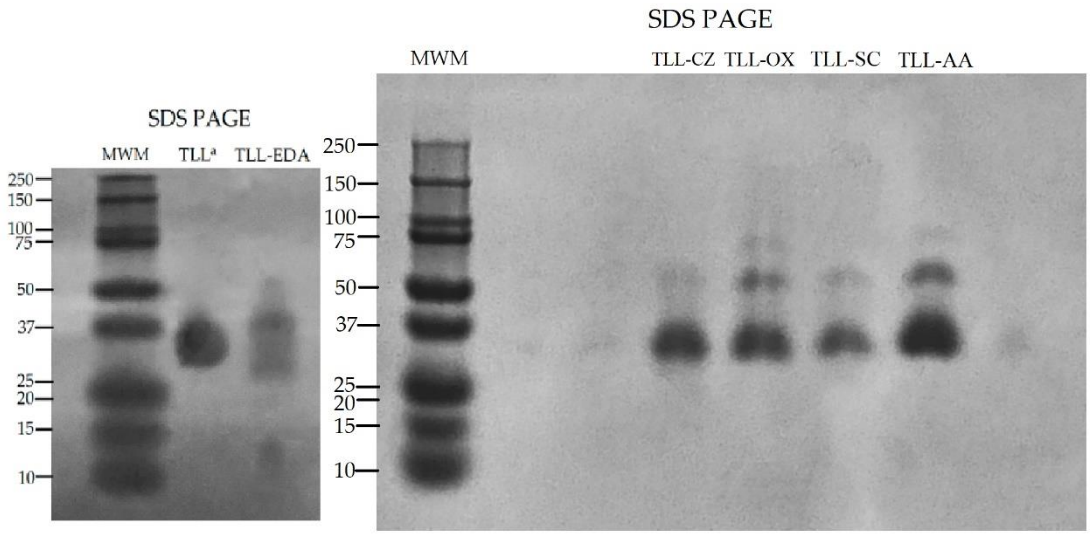

Figure S1. SDS-PAGE experiment used 6% and 12% polyacrylamide gels for concentration and resolution. Sample treatment using hydrazide compounds: TLL[a], TLL-EDA, TLL-CZ, TLL-OX, TLL-SC, TLL-AA.

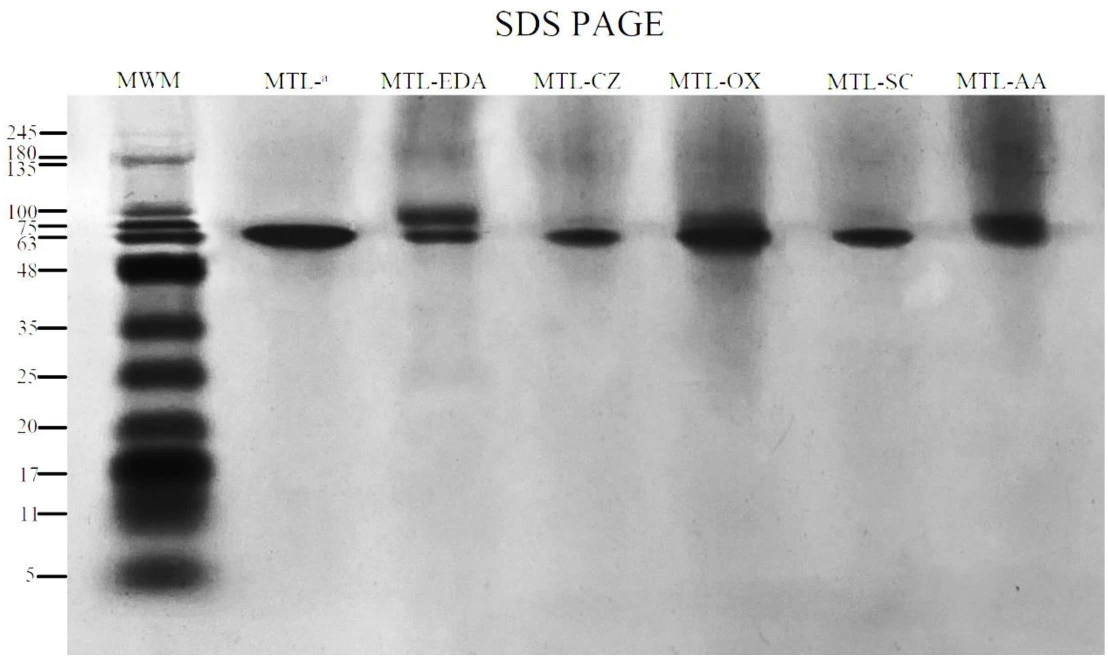

Figure S2. SDS-PAGE experiment used 5% and 10% polyacrylamide gels for concentration and resolution. MTL enzyme modified with hydrazide groups. Includes MTL[a], MTL-EDA, MTL-CZ, MTL-OX, MTL-SC, and MTL-AA.

Table S4. SDS-PAGE summary of modified enzymes, showing the relative molecular weight (Mw), aggregation states, and their relative intensities (Supporting material **Figure S1** and **Figure S2**).

Enzyme	Apparent form	Mw (kDa)	Relative intensity
TLL	*Monomer*	≈31.7	1.00
TLL-EDA	*Monomer*	≈32.5	1.00
TLL-CZ	*Monomer*	≈34.1	0.82
	Dimer	≈63.8	0.18
TLL-OX	*Monomer*	≈34.6	0.60
	Dimer	≈63.8	0.34
	Trimer	≈89.6	0.07
TLL-SC	*Monomer*	≈35.6	0.86
	Dimer	≈66.5	0.14
TLL-AA	*Monomer*	≈35.9	0.80
	Dimer	≈72.2	0.18
	Trimer	≈103	0.03
MTL	*Monomer*	≈62.6	1.00
MTL-EDA	*Monomer*	≈63.9	1.00
MTL-CZ	*Monomer*	≈63.5	1.00
MTL-OX	*Monomer*	≈63.8	1.00
MTL-SC	*Monomer*	≈64.3	1.00
MTL-AA	*Monomer*	≈66.5	1.00

3. Lid dynamics study (TLL)

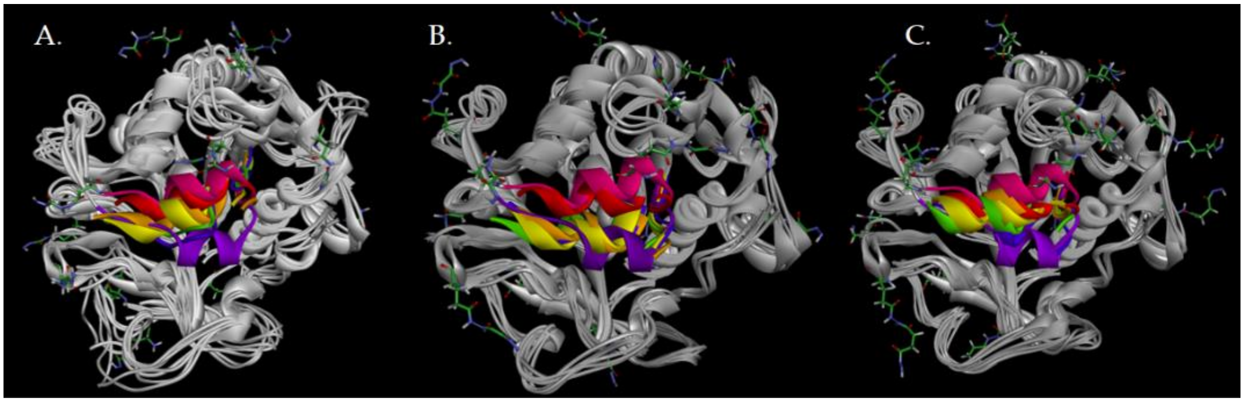

Figure S3. Lid dynamics study of TLL-CZ (A), TLL-OX (B), and TLL-SC (C). They were starting from an open conformation to a more closed one (red 0 ns, orange 18 ns, yellow 43 ns blue 92 ns green 100 ns). PDB structures of TLL open (pink) and close (purple) conformation were aligned as a reference.

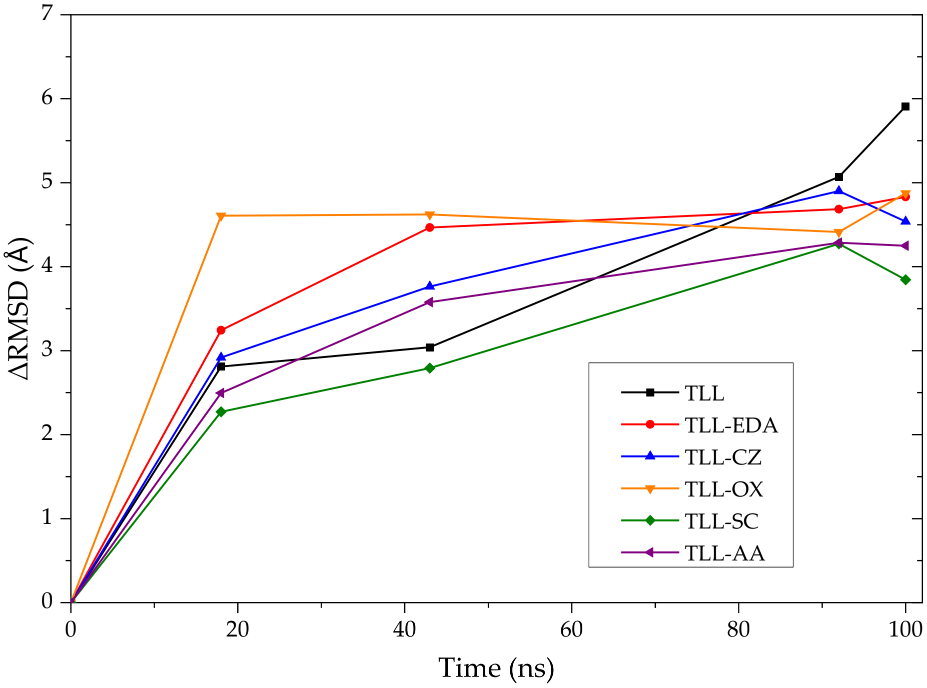

Figure S4. Time evolution of the RMSD variation (ΔRMSD) of the lid region for TLL and its chemically modified variants during molecular dynamics simulations (0–100 ns), calculated relative to the initial open (active) conformation. The trajectories illustrate the conformational transition toward the closed (inactive) state. Color coding: TLL-WM (black), TLL-EDA (red), TLL-CZ (blue), TLL-OX (orange), TLL-SC (green), and TLL-AA (violet).

4. Characterization of modified versions of TLL and MTL

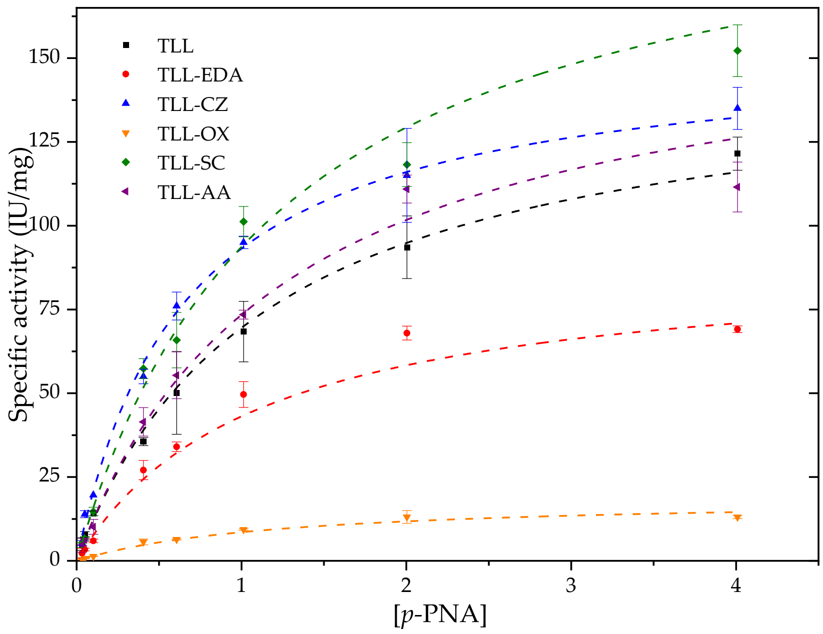

Figure S5. Plot of Michaelis-Menten curves for lipases modified with dihydrazides (TLL-CZ, TLL-OX, TLL-SC, TLL-AA), with EDA (TLL-EDA), and unmodified (TLL-SM). The data is based on the catalytic hydrolysis of *p*-NP into *p*-NPA at 25 °C, pH 7.0, and 0.01% CTAB.

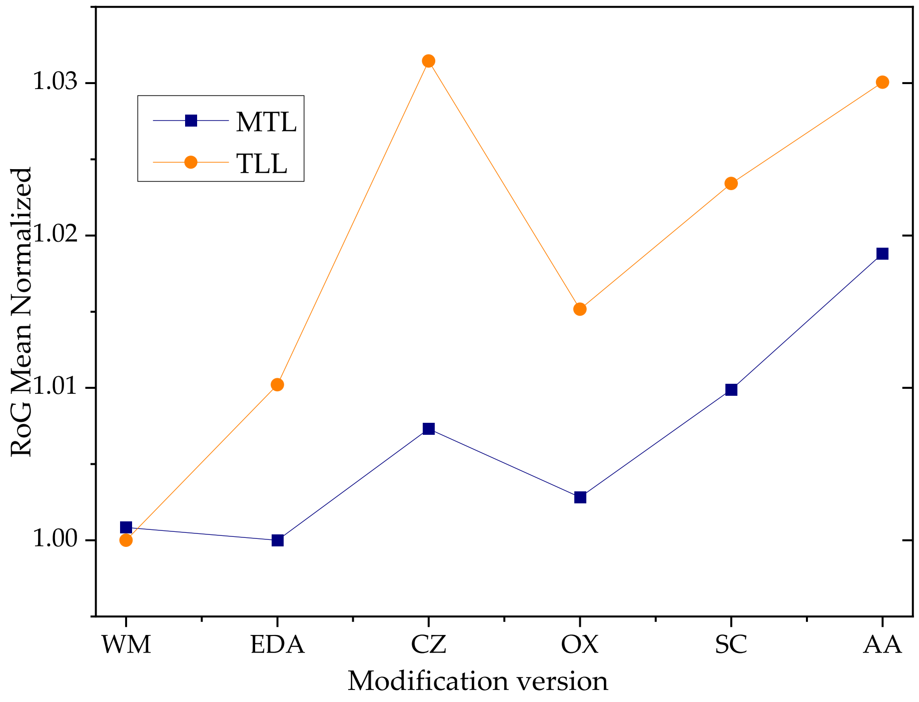

Figure S6. Mean radius of gyration according to the modifier used: WM (unmodified), EDA (reference), CZ, OX, SC, and AA. Orange represents TLL and blue represents MTL.

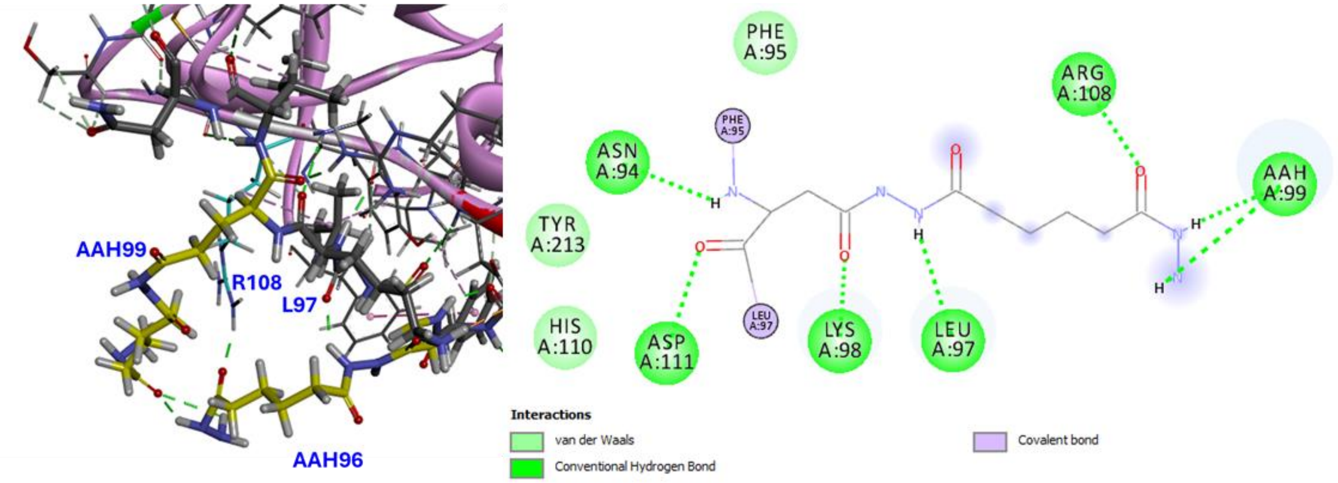

Figure S7. (Left panel) Three-dimensional interaction diagram depicting the local interaction network involving residue AAH96 and its neighboring residues AAH99 and R108, which collectively constrain lid movement and stabilize the open conformation. (Right panel) Two-dimensional interaction diagram showing the interactions of residue AAH96 with neighboring residues, particularly AAH99 and R108, which contribute to restricting lid mobility.

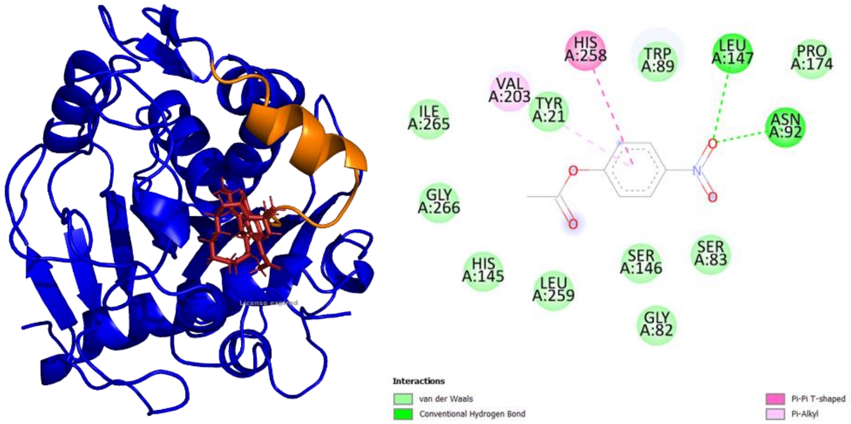

Figure S8. Interactions between the ligand and TLL are illustrated in 2D, revealing van der Waals forces (green), conventional hydrogen bonds (green), Pi–Pi T-shaped interactions (purple), and Pi-Alkyl contacts (pink). Surrounding residues, including those near the active site, are identified based on their proximity and contribution to ligand stabilization.

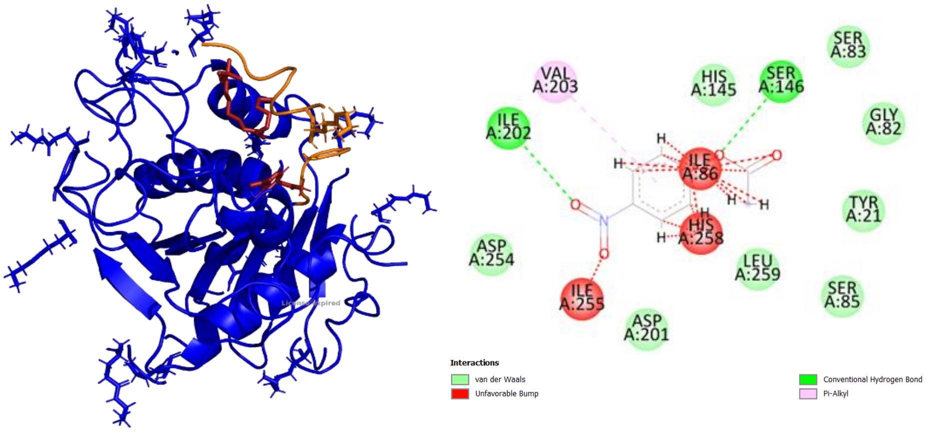

Figure S9. 2D schematic representation of the molecular interactions between the ligand and the TLL-EDA variant, highlighting hydrogen bonds, van der Waals forces, and aromatic interactions within the binding pocket.

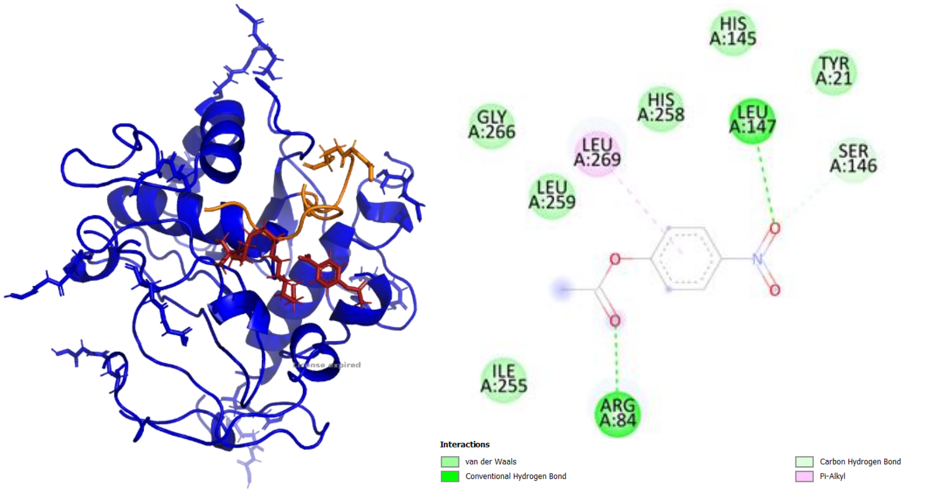

Figure S10. 2D schematic representation of the molecular interactions between the ligand and the TLL-CZ variant, highlighting hydrogen bonds, van der Waals forces, and aromatic interactions within the binding pocket.

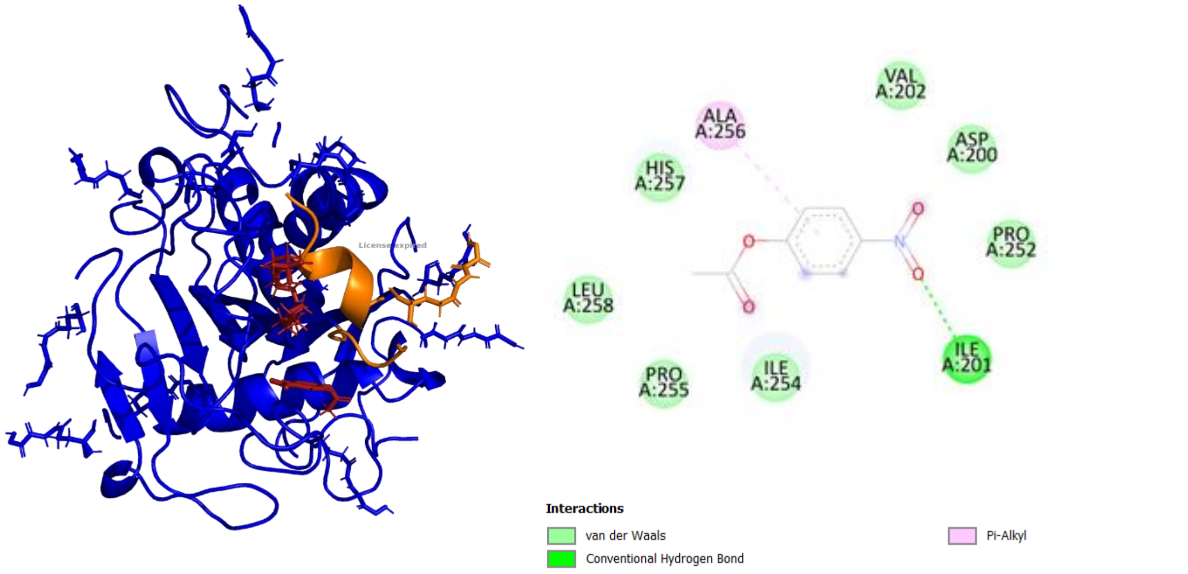

Figure S11. 2D schematic representation of the molecular interactions between the ligand and the TLL-OX variant, highlighting hydrogen bonds, van der Waals forces, and aromatic interactions within the binding pocket.

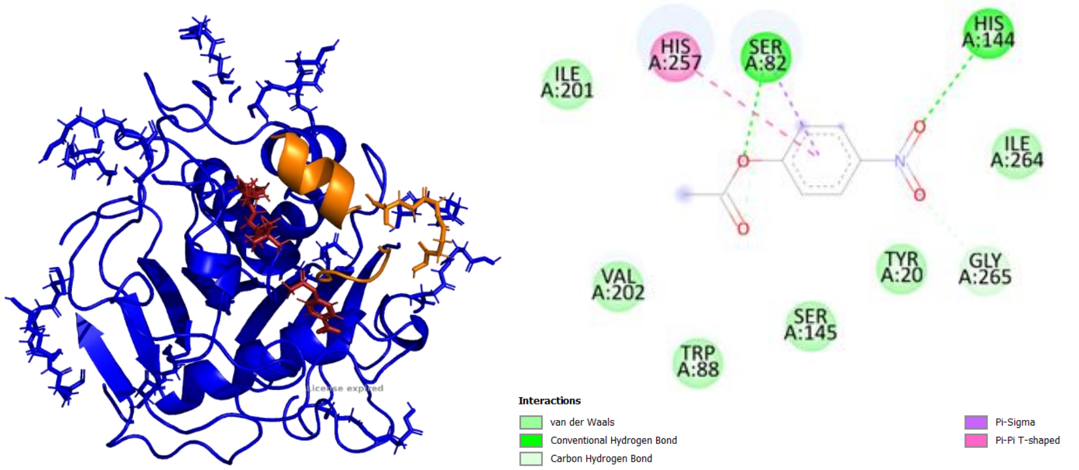

Figure S12. 2D schematic representation of the molecular interactions between the ligand and the TLL-SC variant, highlighting hydrogen bonds, van der Waals forces, and aromatic interactions within the binding pocket.

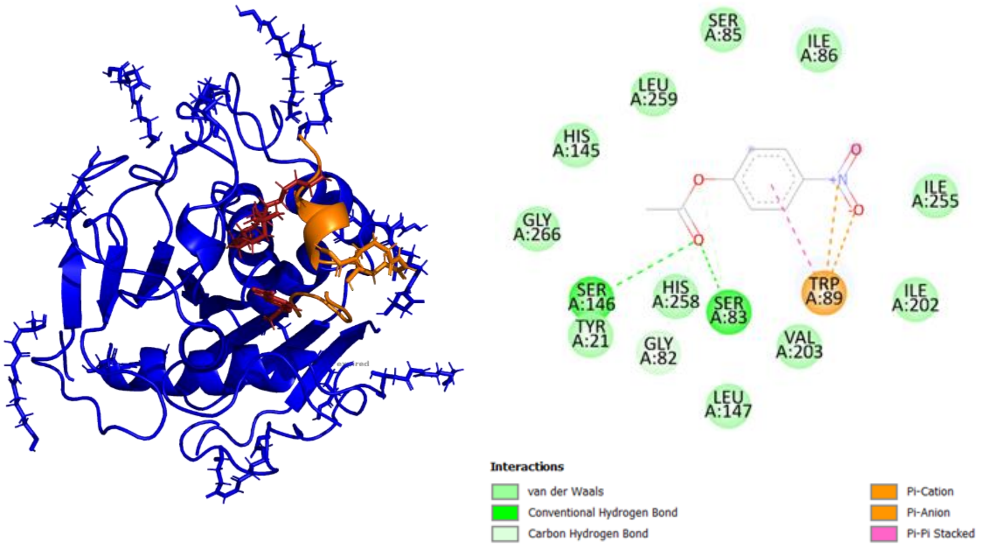

Figure S13. 2D schematic representation of the molecular interactions between the ligand and the TLL-AA variant, highlighting hydrogen bonds, van der Waals forces, and aromatic interactions within the binding pocket.

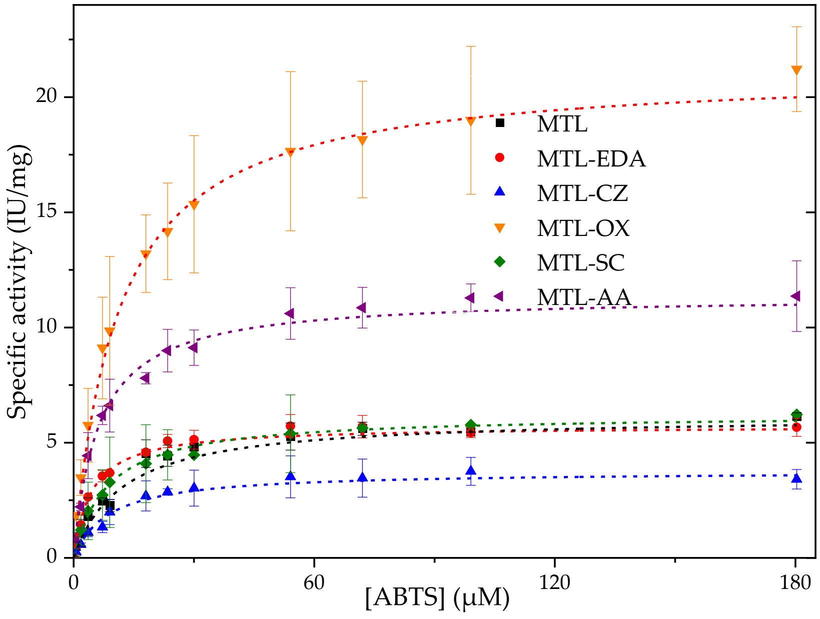

Figure S14. Michaelis-Menten plot for modified, reference, and non-modified laccases. ABTS was used as a substrate, and reactions were carried out at 25 °C and pH 5.0.

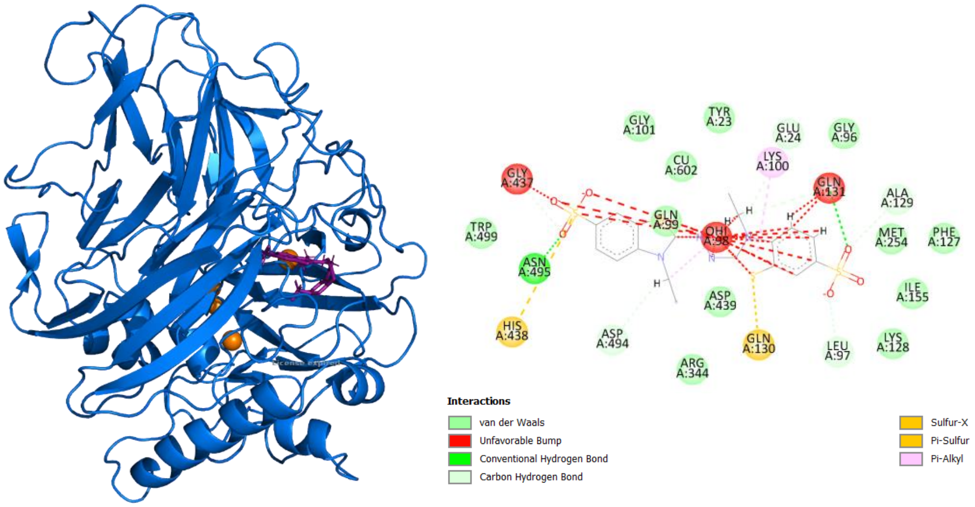

Figure S15. 2D interaction diagram illustrating the molecular contact between the ligand ABTS and MTL, emphasizing key hydrogen bonds, hydrophobic interactions, and steric effects within the active site.

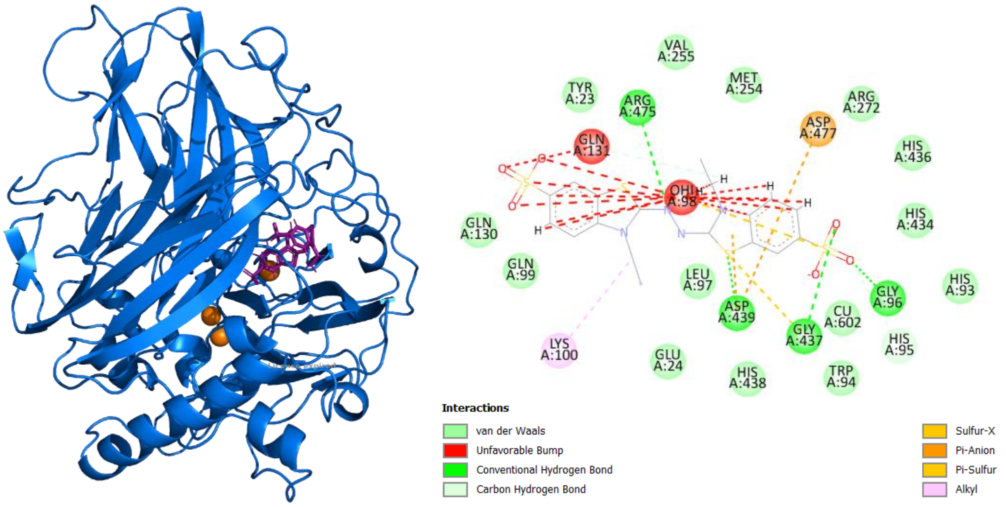

Figure S16. 2D interaction diagram illustrating the molecular contact between the ligand ABTS and MTL-EDA, emphasizing key hydrogen bonds, hydrophobic interactions, and steric effects within the active site.

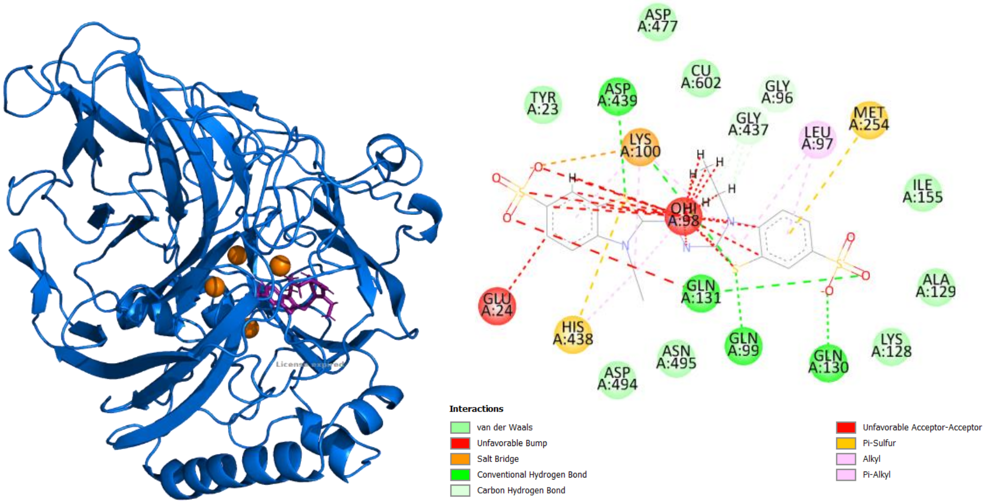

Figure S17. 2D interaction diagram illustrating the molecular contact between the ligand ABTS and MTL-CZ, emphasizing key hydrogen bonds, hydrophobic interactions, and steric effects within the active site.

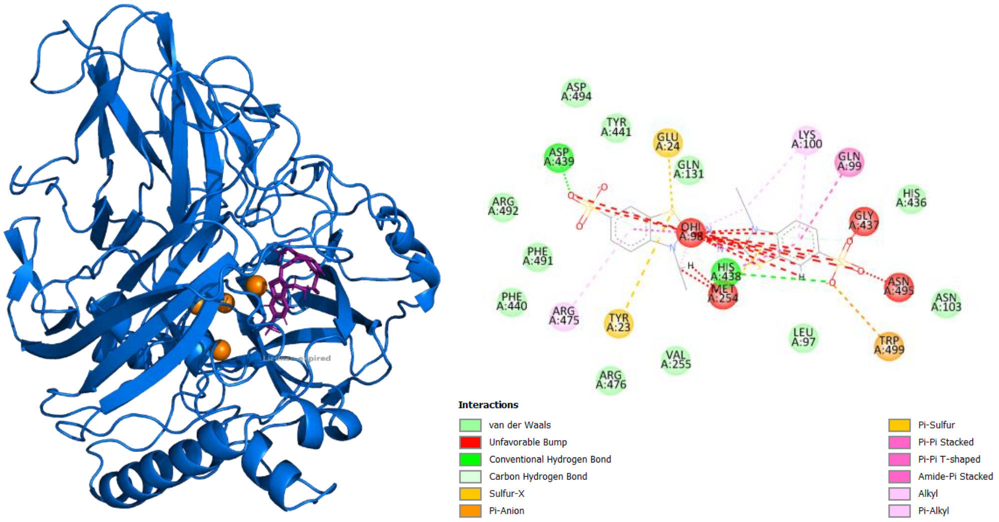

Figure S18. 2D interaction diagram illustrating the molecular contact between the ligand ABTS and MTL-OX, emphasizing key hydrogen bonds, hydrophobic interactions, and steric effects within the active site.

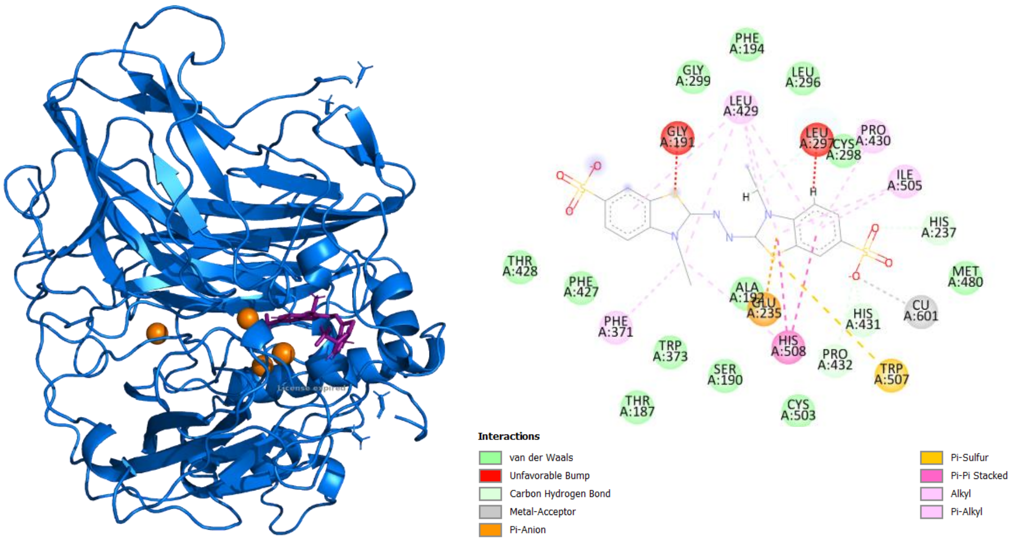

Figure S19. 2D interaction diagram illustrating the molecular contact between the ligand ABTS and MTL-SC, emphasizing key hydrogen bonds, hydrophobic interactions, and steric effects within the active site.

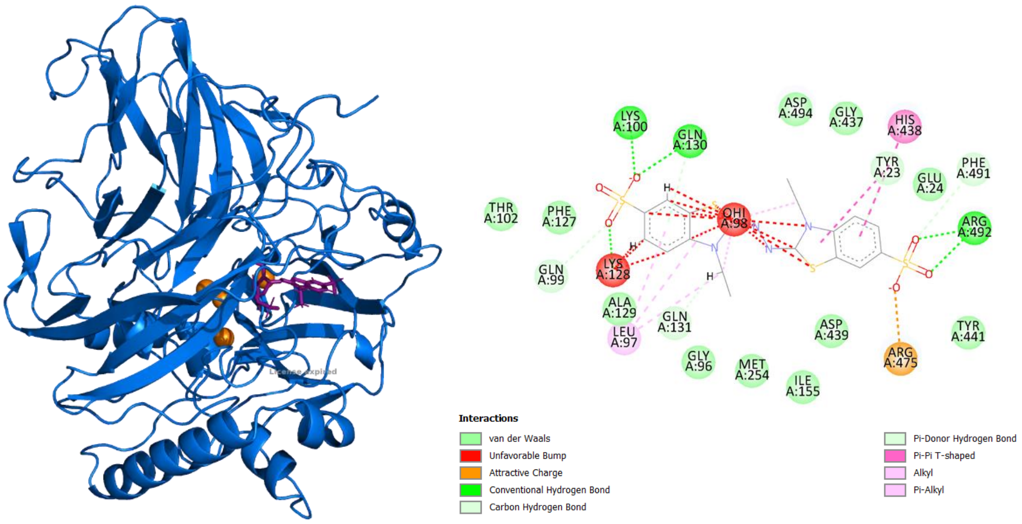

Figure S20. 2D interaction diagram illustrating the molecular contact between the ligand ABTS and MTL-AA, emphasizing key hydrogen bonds, hydrophobic interactions, and steric effects within the active site.

5. Thermal and organic solvent stability of different enzyme variants

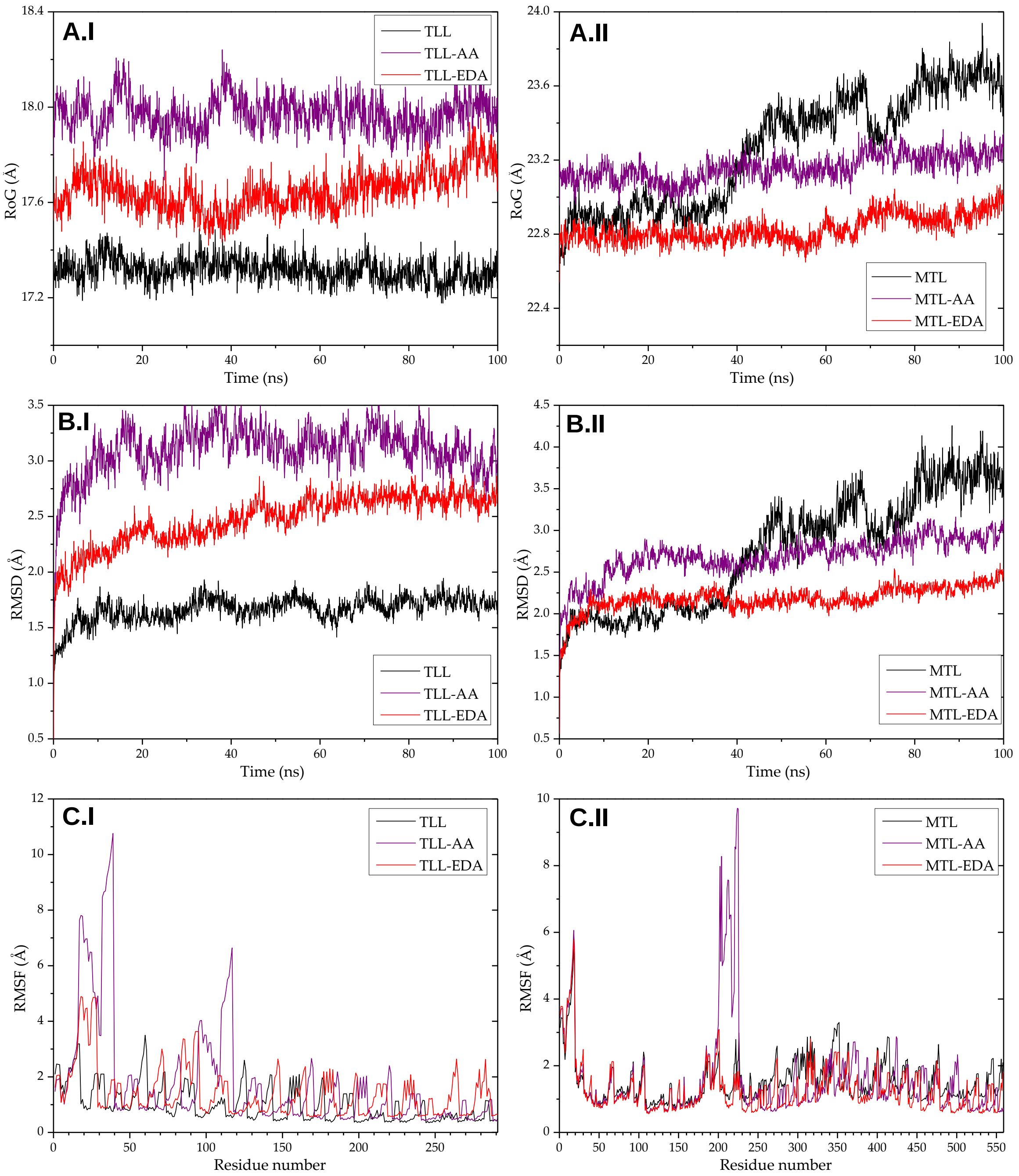

Figure S21. (Panel **A**) radius of gyration (RoG), (Panel **B**) Root mean square deviation (RMSD) and (Panel **C**) root mean square fluctuation (RMSF) values for TLL (I) and MTL (II) and modified versions generated after 100-ns molecular dynamics simulations at 70°C. Without modification (black line); AA-modified (purple line) and EDA-modified (red line).

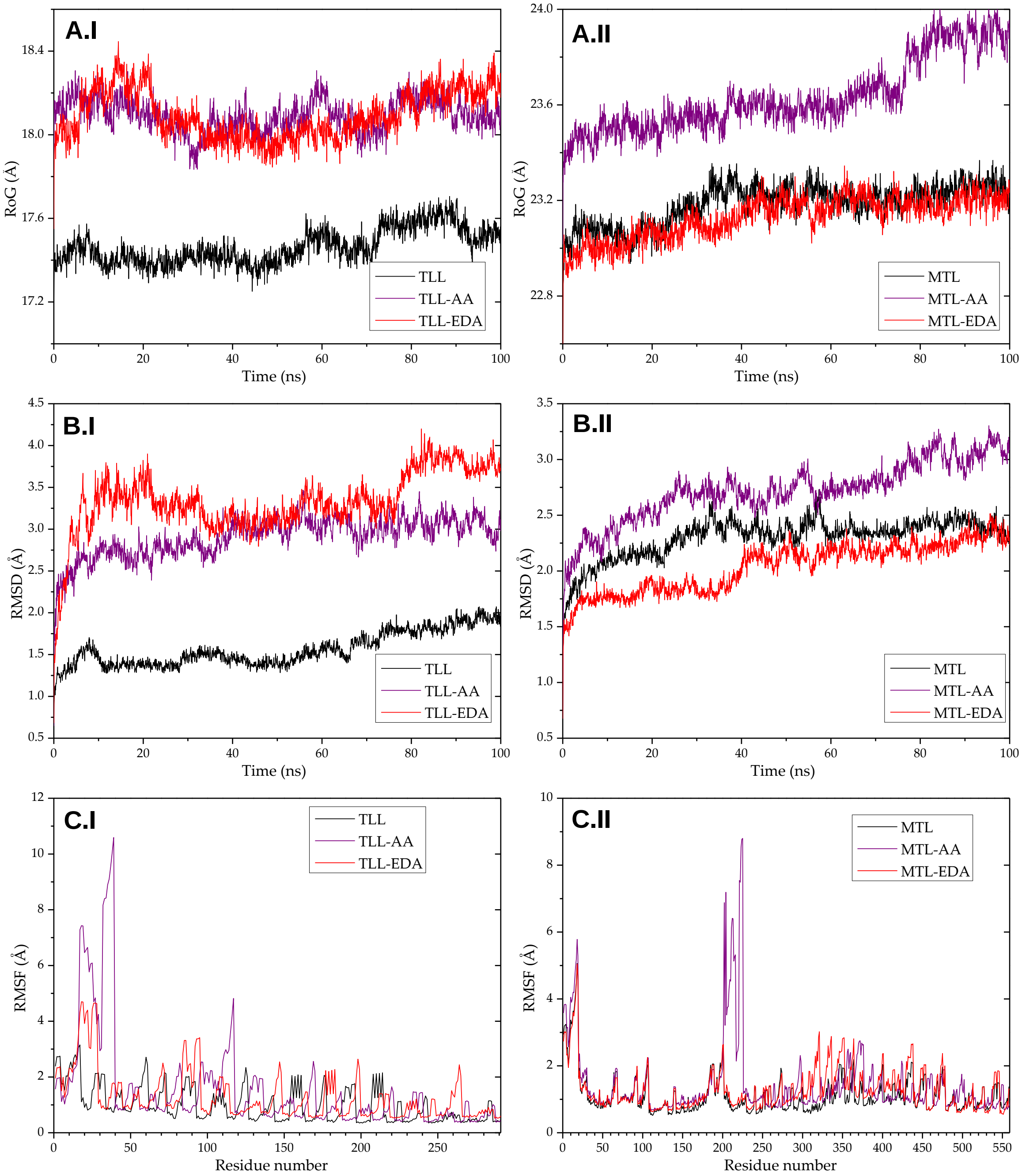

Figure S22. (Panel A) radius of gyration (RoG), (Panel B) Root mean square deviation (RMSD) and (Panel C) root mean square fluctuation (RMSF) values for TLL (I) and MTL (II) and modified versions generated after molecular dynamics simulations at 50 % (v/v) THF, 25°C in water. Without modification (black line); AA-modified (purple line) and EDA-modified (red line).

6. Preliminary assessment of biotechnological applications using enzyme variants

Table S5. FAEE production by TLL, TLL-EDA, and TLL-AA was evaluated under the conditions described in Section 3.11.1. Quantification of fatty acid ethyl esters (FAEEs) was performed by FTIR-ATR using a PCA-based method, with a calibration curve generated from mixtures of oil and the corresponding ethyl esters.

Enzyme	FAEEs (%)
TLL	1.2 ± 0.6
TLL-EDA	3.1 ± 2.4
TLL-AA	2.8 ± 1.8

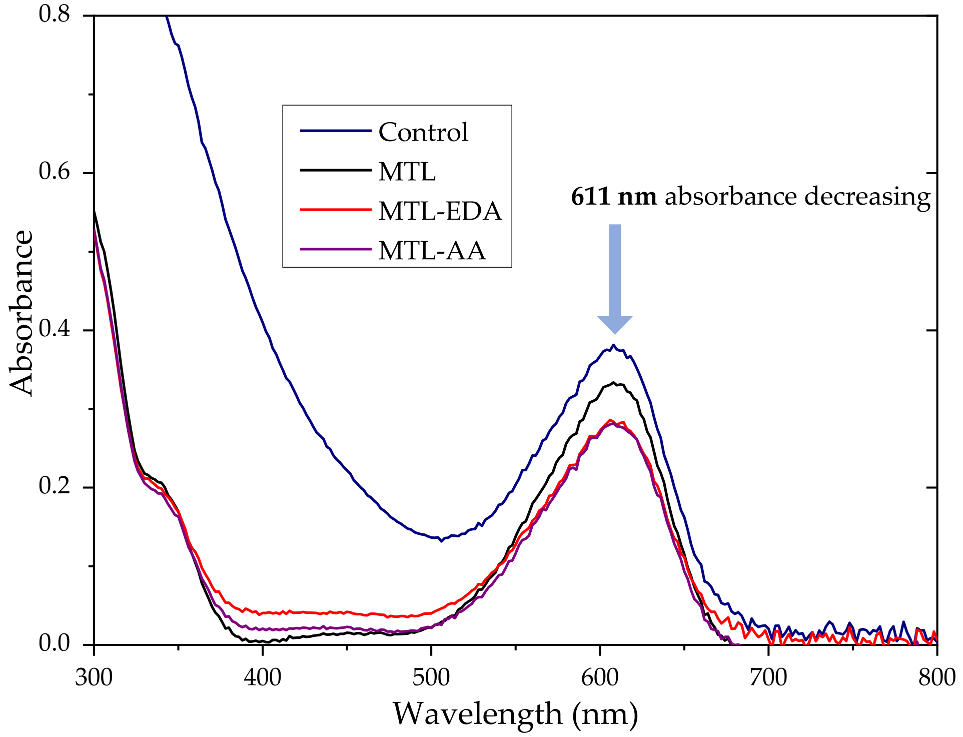

Figure S23. UV-Vis spectra (200–800 nm) of control indigo carmine using enzyme inhibited with sodium azide (blue), and treated with the enzymes: unmodified MTL (black), MTL-EDA (red), and MTL-AA (violet). A moderate decrease of the band with a maximum at 611 nm is observed.

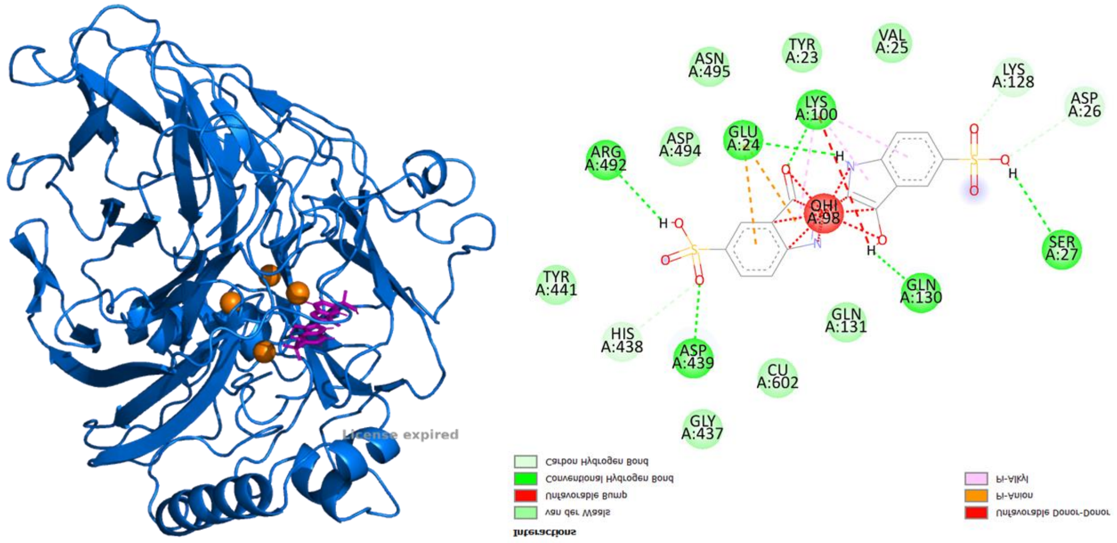

Figure S24. 2D Two-dimensional interaction diagram depicting the binding interactions between MTL and the ligand Indigo Carmine, highlighting key hydrogen bonding, hydrophobic contacts, and steric hindrance effects within the active site.

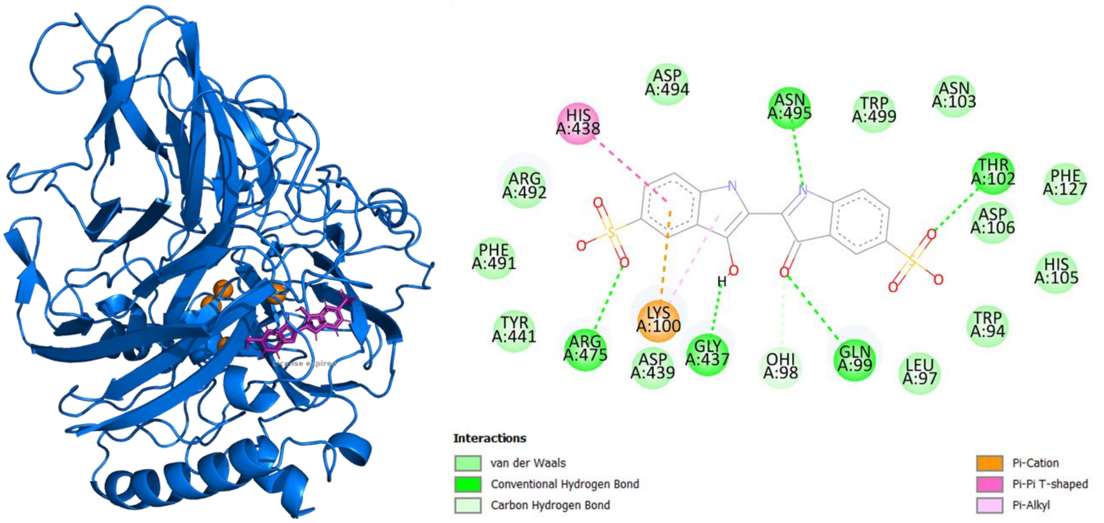

Figure S25. 2D interaction diagram depicting the binding interactions between MTL-EDA and the ligand Indigo Carmine, highlighting key hydrogen bonding, hydrophobic contacts, and steric hindrance effects within the active site.

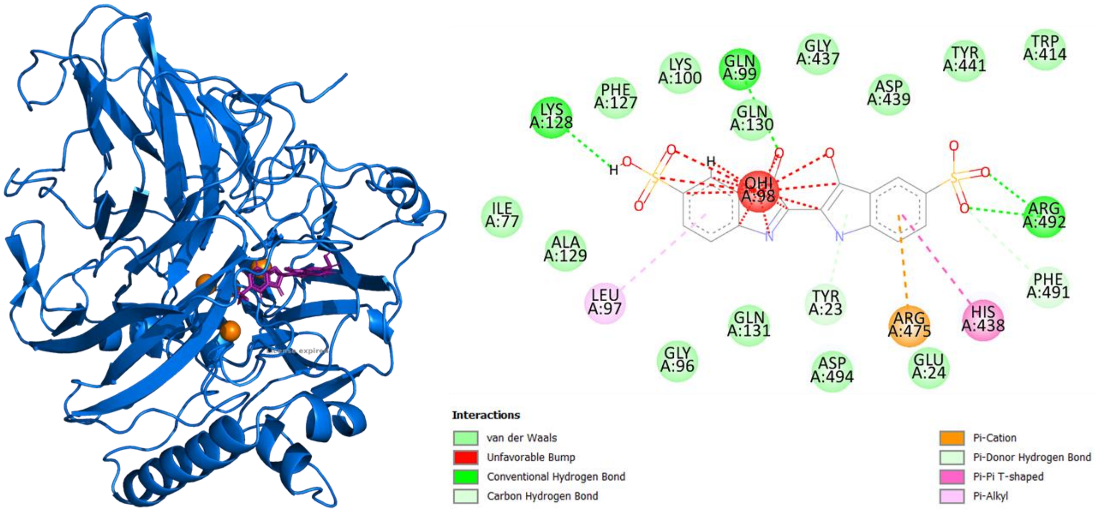

Figure S26. 2D interaction diagram depicting the binding interactions between MTL-AA and the ligand Indigo Carmine, highlighting key hydrogen bonding, hydrophobic contacts, and steric hindrance effects within the active site.

7. Experimental procedures details

7.1 Determination of percentage of modification using TNBS assay.

The modification percentage was determined using the 2,4,6-trinitrobenzene sulfonic acid (TNBS) method, which quantifies free amines or hydrazides [44,57,58]. Protein samples in sodium borate buffer (pH 8.5) were incubated with 0.008% (w/v) TNBS at 800 rpm, 30°C for 40 min. Before the measurement, the reaction was quenched with sodium carbonate buffer at pH 10.8. The respective groups were quantified by the increase of the 2,4,6-trinitrobenzene amine (TNB-NH) or 2,4,6-trinitrobenzene hydrazide (TNB-NH-NH-) derivatives under monitoring at 420 nm (for EDA derivative) and 500 nm (for hydrazide derivative) (see **Figure S27.** Calibration plots obtained from the TNBS assay for amine groups (black), using bovine serum albumin (BSA) as standard, and hydrazide groups (red), using octanoic hydrazide as standard. The absorbance was measured at 420 nm (amines) and 500 nm (hydrazides) after reaction with 0.008% (w/v) TNBS at 30 °C for 40 min. Regression lines and corresponding equations are shown in each panel. Analytical parameters are summarized in **Table S6. and Figure S28**, supplementary information).

Calibration curves were generated using bovine serum albumin (BSA) and octanoic hydrazide as standards for amine and hydrazide quantification, respectively. **Figure S27** shows the linear regression plots obtained for both systems, and **Table S6** summarizes the corresponding calibration parameters, including R^2, adjusted R^2, standard error (S), linear range, and detection and quantification limits.

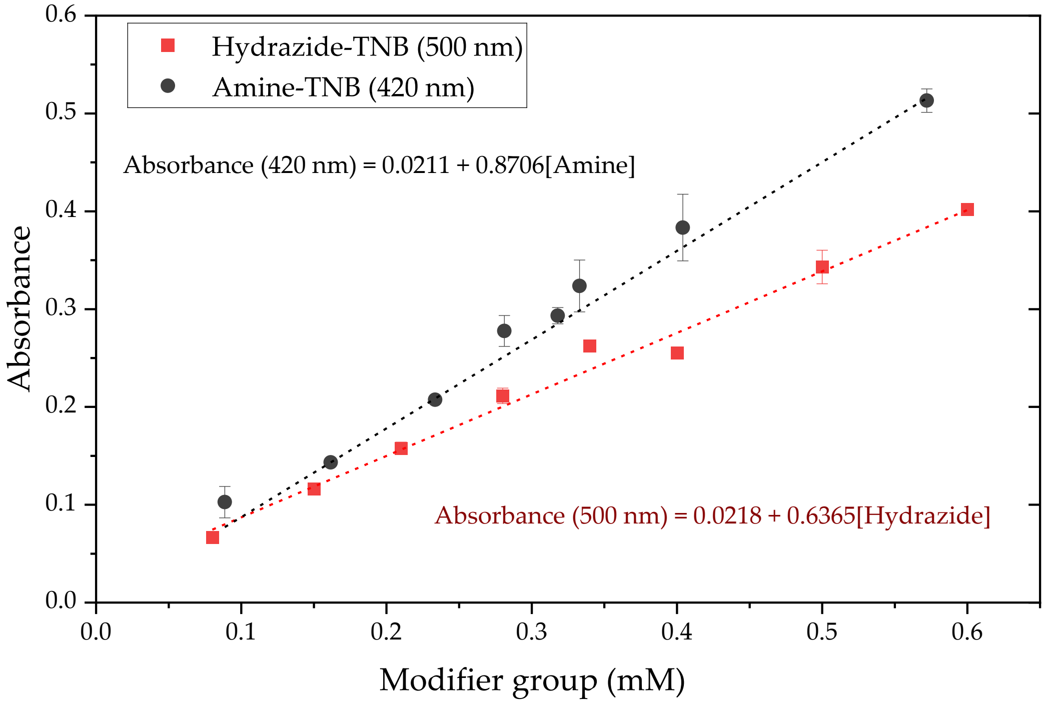

Figure S27. Calibration plots obtained from the TNBS assay for amine groups (black), using bovine serum albumin (BSA) as standard, and hydrazide groups (red), using octanoic hydrazide as standard. The absorbance was measured at 420 nm (amines) and 500 nm (hydrazides) after reaction with 0.008% (w/v) TNBS at 30 °C for 40 min. Regression lines and corresponding equations are shown in each panel. Analytical parameters are summarized in **Table S6**.

Table S6. Parameters of the calibration curves obtained from the TNBS assay for the quantification of amine and hydrazide groups. Values correspond to the linear regression models shown in **Figure S27**.

Parameter	TNBS Curves		BSA Protein Curve
	Amines (420 nm)	Hydrazides (500 nm)	
Regression equation	0.0211 + 0.8706 [Amine]	0.0218 + 0.6365 [Hydrazide]	0.5498 + 1.1996 [BSA]
R^2	0.994	0.985	0.991
Adjusted R^2	0.992	0.982	0.988
Linear range	0.09 -0.57 (mM)	0.08 - 0.60 (mM)	0.08 - 1.00 (mg/mL)
$S_{y/x}$	0.0127	0.0164	0.052
LOD (mM)	0.01 (mM)	0.08 (mM)	0.11
LOQ (mM)	0.15 (mM)	0.25 (mM)	0.42

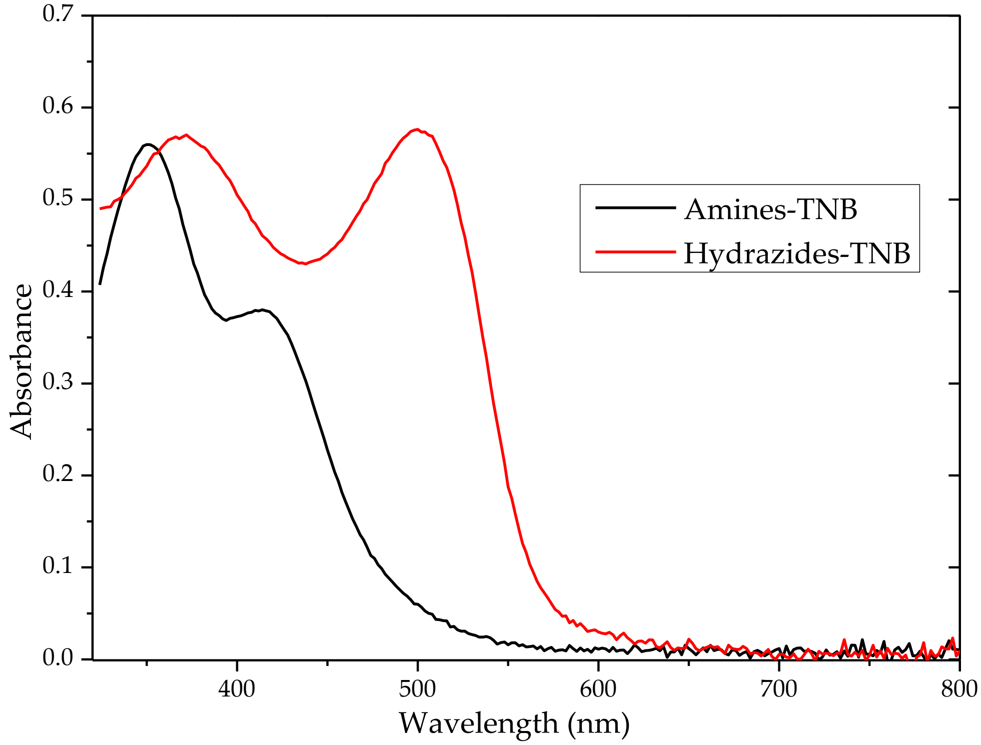

Figure S28. Absorbance spectrum of amine and hydrazide compounds in the presence of TNBS.

To correlate modified groups with enzyme quantity (µmol/mg), protein content in each sample was measured via the Bradford method, and µmol of modified groups (amine or hydrazide) per mg of enzyme was determined. The modification percentage was calculated using Equation 1 (Eq S1), comparing the modified enzyme (EM) with the unmodified enzyme (E0), and normalized to available carboxyl groups to be modified (32 for TLL and 42 for MTL).

$$\% \, modification = \frac{[NH - TNB]_{EM} - [NH - TNB]_{E0}}{[CO_2^-]_0} x100 \quad \text{(Eq S1)}$$

Where $[NH\text{-}TNB]_{EM}$ is the amount of amines or hydrazides incorporated in the enzyme, $[NH\text{-}TNB]_{E0}$ is the groups of the enzyme before modification (this number for modification with hydrazides is zero), and $[CO_2^-]_0$ is the amount of available carboxyl groups.

7.2 SDS-PAGE densitometry analysis

Densitometric analysis was performed by scanning gels in transmission mode (automatic gain disabled) [60]. Grayscale and optical density calibrations were performed in ImageJ using a step tablet and the Rodbard function, respectively [60]. Band profiles were generated from selected lanes using the rectangular selection tool with the Plot Lanes function, and peak areas were quantified using the Wand tool (ImageJ). This analysis was used to estimate the relative concentrations of enzyme aggregates reported in Table S4 (Supporting Information).
